# Discerning morpho-anatomical, physiological and molecular multiformity in cultivated and wild genotypes of lentil with reconciliation to salinity stress

**DOI:** 10.1371/journal.pone.0177465

**Published:** 2017-05-25

**Authors:** Dharmendra Singh, Chandan Kumar Singh, Shanti Kumari, Ram Sewak Singh Tomar, Sourabh Karwa, Rajendra Singh, Raja Bahadur Singh, Susheel Kumar Sarkar, Madan Pal

**Affiliations:** 1 Division of Genetics, ICAR-Indian Agricultural Research Institute, New Delhi, India; 2 Division of Soil Science and Agricultural Chemistry, ICAR-Indian Agricultural Research Institute, New Delhi, India; 3 ICAR-All India Co-ordinated Research Project on Salt affected Soil and Saline Use in Irrigation Water in Agriculture, Raja Balwant Singh College, Bichpuri, Agra, India; 4 ICAR-National Research Centre on Plant Biotechnology, Pusa Campus, New Delhi, India; 5 Division of Plant Physiology, ICAR-Indian Agricultural Research Institute, New Delhi, India; 6 ICAR-Indian Agricultural Statistical Research Institute, New Delhi, India; Estacion Experimental del Zaidin, SPAIN

## Abstract

One hundred and sixty two genotypes of different *Lens* species were screened for salinity tolerance in hydroponics at 40, 80 and 120 mM sodium chloride (NaCl) for 30 d. The germination, seedling growth, biomass accumulation, seedling survivability, salinity scores, root and shoot anatomy, sodium ion (Na^+^), chloride ion (Cl^-^) and potassium ion (K^+^) concentrations, proline and antioxidant activities were measured to evaluate the performance of all the genotypes. The results were compared in respect of physiological (Na^+^, K^+^ and Cl^-^) and seed yield components obtained from field trials for salinity stress conducted during two years. Expression of salt tolerance in hydroponics was found to be reliable indicator for similarity in salt tolerance between genotypes and was evident in saline soil based comparisons. Impressive genotypic variation for salinity tolerance was observed among the genotypes screened under hydroponic and saline field conditions. Plant concentrations of Na^+^ and Cl^-^ at 120 mM NaCl were found significantly correlated with germination, root and shoot length, fresh and dry weight of roots and shoots, seedling survivability, salinity scores and K^+^ under controlled conditions and ranked the genotypes along with their seed yield in the field. Root and shoot anatomy of tolerant line (PDL-1) and wild accession (ILWL-137) showed restricted uptake of Na^+^ and Cl^-^ due to thick layer of their epidermis and endodermis as compared to sensitive cultigen (L-4076). All the genotypes were scanned using SSR markers for genetic diversity, which generated high polymorphism. On the basis of cluster analysis and population structure the contrasting genotypes were grouped into different classes. These markers may further be tested to explore their potential in marker-assisted selection.

## Introduction

Salinity affected arable area globally is around 100 million ha worldwide, and is increasing day by day [1]. Salinity in soil or water is one of the major threats, especially in arid and semi-arid ecology, as it severely limits plant growth and productivity, primarily due to ion toxicity and reducing osmotic potential. Lentil is sensitive to salinity which is magnified in arid and semi-arid regions. Soil and water management approaches may be adopted for amelioration of soil salinity in these areas, but these approaches are expensive for small holding farmers. Therefore, development of salt-tolerant genotypes is the most appropriate and cost effective strategy, for stabilizing yield in salinity affected areas.

Various traits like germination, visual salt injury, seedling survival, reduction in seedling growth, biomass accumulation, Na^+^, Cl^-^, K^+^ contents, proline, Hydrogen peroxide (H_2_O_2_) production and antioxidant activities have been used in the past for evaluation of salt tolerance in various crops. Germination, seedling survival, growth and biomass were found to be affected under salinity stress in crop plants [2–5]. The response of plants to salinity varies depending upon the growth, developmental stages and duration of exposure to salinity stress [6]. Germination stage was found to be less sensitive to salinity than early vegetative growth stage [7, 8]. However, the reproductive phase has been reported to be more sensitive than vegetative one [9]. The screening of genotypes at seedling stage is easier than at vegetative or reproductive stages [10] and early growth stages have shown better plant’s response to salinity [11], therefore, initial screening for salt tolerance at seedling stage is considered more important. Salinity also affects anatomical structures to a great extent. A major reduction in vascular tissue mass was observed in roots of *Chloris gayana* Kunth under salinity stress [12]. Dua and Sharma reported that reduction in growth was accompanied with higher accumulation of Na^+^ and Cl^-^ in tissues when exposed to high NaCl concentration [13]. Entry of both Na^+^ and Cl^-^ into the cells leads to severe ion imbalance and excess uptake might cause significant physiological disorder (s). Plants can reduce Na^+^ and Cl^-^ toxicity through regulation of Na^+^ and Cl^-^ uptake, xylem loading, retrieval from xylem, extrusion from roots and intracellular compartmentation in the vacuole and osmolyte accumulation such as proline. The controlled uptake of Na^+^ and/ or Cl^-^ is considered to be the key mechanism contributing salt tolerance. High Na^+^ and Cl^-^ concentrations enhances production of ROS (reactive oxygen species) and inhibits uptake of K^+^ ions, which contributes to low crop productivity and may even lead to death of plants [14, 15]. The accumulation of proline and antioxidant enzymes like superoxide dismutase (SOD), catalase (CAT), ascorbate peroxidase (APX) and Glutathione peroxidase (GPX) are efficiently involved in scavenging ROS (reactive oxygen spices) produced during salt stress conditions, and also act as an important tolerance mechanism against oxidative stress in plants [16–18]. Despite a number of phenotypic investigations, the molecular mechanisms for biochemical and physiological traits are still limited. There are few kinases, phosphatases and transcription factors involved in plants that are associated with salinity tolerance [19]. These processes interact with one another to impart resistance to salt at molecular or whole plant level [20–21].

Evaluation of plants to salt tolerance in hydroponics [22–24] and field conditions [25, 26] has been well documented. The hydroponic technique has unconditional advantages over field screening, because the saline conditions in the field may be heterogenous and environmental factors may mask the expression of salt tolerance. However, initial screening in hydroponic and later conferring in saline field conditions could be the best strategy for accurate phenotyping against salt tolerance.

Molecular assortment of morpho-physiologically characterized genotypes can directly help lentil breeders to choose contrasting parents for hybridization and incorporating salinity tolerance. Different molecular markers, including simple sequence repeats (SSRs), amplified fragment length polymorphism (AFLPs) and inter simple sequence repeats (ISSRs) have been successfully used for identification of diversity among the genotypes and gene/QTL analyses in lentil [27–30]. Simple sequence repeat markers have extensively been used for the assessment of genetic diversity in lentil [31–33] as these markers have high efficiency, easy to use, high reproducibility, co-dominance behaviour and high polymorphism. To our knowledge, no detailed information is available on genetic diversity among the cultivated and wild species of lentil with reconciliation to salinity stress using morpho-anatomical and physiological traits and molecular markers. Therefore, the present study was planed (i) to explore the magnitude of variation for tolerance to salinity by screening a large number of genotypes using morpho-physiological criteria, (ii) to compare the morpho-anatomical and physiological responses of most diverse genotypes to salinity under hydroponics and field, (iii) to rank the genotypes for response to salinity both under hydroponics and saline field conditions, iv) to assess the mechanism of salt tolerance, and iv) to assort genetically diverse genotypes using SSR markers.

## Materials and methods

### Plant materials

Seeds of 162 accessions (120 germplasm/ landraces and 42 wild accessions), originated from 14 countries were procured from **International Center for Agricultural Research in the Dry Areas** (ICARDA). The wild accessions included *L*. *orientalis* (5), *L*. *odomensis* (14), *L*. *nigricans* (4), *L*. *ervoides* (16) and *L*. *lamottei* (3) (Table 1). The first two wild accessions belong to the primary gene pool, while the last three belong to the secondary/tertiary gene pool.

**Table 1:** Name, origin and salt tolerance category of genotypes used in the study.

S. No.	Genotype	Origin	Type	SR	S.No.	Genotype	Origin	Type	SR
1	121–12	India	GC	S	82	ILWL-203	Turkey	Wild	T
2	1220–11	India	BL	S	83	ILWL-227	Syria	Wild	S
3	210–11	India	BL	S	84	ILWL-238	Syria	Wild	S
4	330–12	India	GC	S	85	ILWL-29	Spain	Wild	S
5	BM-4	Bangladesh	Cult.	S	86	ILWL-292	Turkey	Wild	S
6	DPL-62	India	Cult.	S	87	ILWL-320	Turkey	Wild	S
7	E-153	India	GC	S	88	ILWL-334	Jordan	Wild	S
8	FLIP-96-51	ICARDA	GC	S	89	ILWL-340	Jordan	Wild	MT
9	IG-109039	ICARDA	GC	S	90	ILWL-35	Turkey	Wild	MT
10	IG-111991	ICARDA	LR	S	91	ILWL-350	Syria	Wild	S
11	IG-111996	ICARDA	LR	S	92	ILWL-361	Syria	Wild	S
12	IG-112078	ICARDA	LR	S	93	ILWL-362	Syria	Wild	S
13	IG-11210	ICARDA	LR	S	94	ILWL-398(A)	Lebanon	Wild	MT
14	IG-112128	ICARDA	LR	S	95	ILWL-401	Lebanon	Wild	S
15	IG-112131	ICARDA	LR	S	96	ILWL-415	Syria	Wild	S
16	IG-112137	ICARDA	LR	S	97	ILWL-418	Syria	Wild	MT
17	IG-116551	ICARDA	LR	S	98	ILWL-428	Spain	Wild	T
18	IG-129185	ICARDA	LR	S	99	ILWL-436	Turkey	Wild	MT
19	IG-129287	ICARDA	LR	S	100	ILWL-438	Turkey	Wild	S
20	IG-129291	ICARDA	LR	S	101	ILWL-44	Slovenia	Wild	S
21	IG-129293	ICARDA	LR	S	102	ILWL-447	Turkey	Wild	S
22	IG-129302	ICARDA	LR	S	103	ILWL-462	Turkey	Wild	MT
23	IG-129309	ICARDA	LR	S	104	ILWL-464	Syria	Wild	MT
24	IG-129313	ICARDA	LR	S	105	ILWL-58	Turkey	Wild	S
25	IG-129315	ICARDA	LR	S	106	ILWL-83	Turkey	Wild	MT
26	IG-129317	ICARDA	LR	MT	107	ILWL-95	Turkey	Wild	T
27	IG-129319	ICARDA	LR	MT	108	IPL-406	India	Cult.	S
28	IG-129560	ICARDA	LR	S	109	JL-3	India	Cult.	S
29	IG-130033	ICARDA	LR	S	110	L-404	India	BL	S
30	IG-130219	ICARDA	LR	S	111	L-4076	India	Cult.	S
31	IG-134342	ICARDA	LR	S	112	L-4078	India	BL	S
32	IG-134356	ICARDA	LR	S	113	L-4147	India	Cult.	S
33	IG-135424	-	Wild	S	114	L-4578	India	BL	S
34	IG-136607	ICARDA	LR	S	115	L-4590	India	Cult.	MT
35	IG-136618	Croatia	Wild	S	116	L-4594	India	Cult.	MT
36	IG-136620	Slovenia	Wild	S	117	L-4602	India	BL	S
37	IG-136637	France	Wild	S	118	L-4603	India	BL	S
38	IG-129304	ICARDA	LR	S	119	L-4618	India	BL	MT
39	IG-5320	ICARDA	LR	MT	120	L-4619	India	BL	MT
40	IG-70174	ICARDA	LR	S	121	L-4620	India	BL	S
41	IG-70230	ICARDA	LR	MT	122	L-4650	India	BL	S
42	IG-71646	ICARDA	LR	S	123	L-4701	India	BL	S
43	IG-71710	ICARDA	LR	MT	124	L-5253	India	BL	S
44	IG-73798	ICARDA	LR	S	125	L-7752	India	BL	MT
45	IG-73802	ICARDA	LR	S	126	L-7818	India	BL	MT
46	IG-73816	ICARDA	LR	S	127	L-7903	India	BL	MT
47	IG-73945	ICARDA	LR	S	128	L-7905	India	BL	MT
48	IG-75920	ICARDA	LR	S	129	L-7920	India	BL	S
49	IG-9	ICARDA	LR	S	130	LC-270-804	India	BL	S
50	IG-936	ICARDA	LR	S	131	LC-282-1077	India	BL	S
51	ILL-10030	ICARDA	GC	S	132	LC-282-1110	India	BL	MT
52	ILL-10031	ICARDA	GC	S	133	LC-282-896	India	BL	S
53	ILL-10032	ICARDA	GC	S	134	LC-284-116	India	BL	S
54	ILL-10034	ICARDA	GC	S	135	LC-284-1209	India	BL	S
55	ILL-10795	ICARDA	GC	S	136	LC-285-1344	India	BL	S
56	ILL-10810	ICARDA	GC	S	137	LC-289-1444	India	BL	S
57	ILL-10820	ICARDA	GC	S	138	LC-289-1447	India	BL	S
58	ILL-10823	ICARDA	GC	S	139	LC-292-1485	India	BL	S
59	ILL-10826	ICARDA	GC	S	140	LC-292-1544	India	BL	S
60	ILL-10827	ICARDA	GC	S	141	LC-292-997	India	BL	S
61	ILL-10835	ICARDA	GC	S	142	LC-300-11	India	BL	MT
62	ILL-10848	Bangladesh	GC	S	143	LC-300-12	India	BL	S
63	ILL-10897	ICARDA	GC	S	144	LC-300-13	India	BL	S
64	ILL-10913	ICARDA	GC	S	145	LC-300-15	India	BL	MT
65	ILL-10921	ICARDA	GC	S	146	LC-300-16	India	BL	S
66	ILL-10967	ICARDA	GC	S	147	LC-300-2	India	BL	S
67	ILL-10969	ICARDA	GC	S	148	LC-300-3	India	BL	S
68	ILL-3829	ICARDA	GC	S	149	LC-300-4	India	BL	S
69	ILL-4605	Argentina	Cult.	S	150	LC-300-6	India	BL	S
70	ILL-6002	ICARDA	GC	S	151	LC-300-7	India	BL	S
71	ILWL-06	Turkey	Wild	S	152	LC-300-8	India	BL	S
72	ILWL-09	Syria	Wild	T	153	LC-300-9	India	BL	MT
73	ILWL-10	-	Wild	S	154	LC-74-1-51	India	BL	S
74	ILWL-125	Syria	Wild	S	155	PDL-1	ICARDA	BL	T
75	ILWL-128	Syria	Wild	S	156	PDL-2	ICARDA	BL	MT
76	ILWL-13	Italy	Wild	MT	157	PL-5	India	Cult.	MT
77	ILWL-133	Syria	Wild	S	158	PSL-1	ICARDA	GC	MT
78	ILWL-137	Syria	Wild	T	159	PSL-9	India	GC	T
79	ILWL-15	France	Wild	MT	160	SEHORE-74-3	India	Cult.	MT
80	ILWL-192	Syria	Wild	S	161	SKL-259	India	BL	S
81	ILWL-20	Palestine	Wild	S	162	WBL-77	India	Cult.	S

SR = Salanity Reaction; GC = Germplasm collection; BL = Breeding Lines; LR = Land races; S = Sensitive; T = Tolerance; MT = Moderately Tolerances in SR

### Evaluation of genotypes under salinity stress

Seeds of lentil were sterilized in sodium hypochloride (12%) for 15min, thereafter were washed three times with distilled water. Twenty seeds of each genotype were placed in plastic petridishes on filter paper wetted with distilled water (control) and other three in salinity levels (0, 40, 80 and 120 mM NaCl)]. The germination was recorded at 12 h interval for 10 days. Seeds were considered germinated when their radicals emerged out and attained 1 mm length. Germination percentage was calculated based on the following formula: Germination per cent = (Total seeds germinated) / (Total seeds sown) x 100. A completely randomized block design was used for each salinity treatment, with three replicates of 15 seeds each.

The hydroponic experiment was conducted at National Phytotron Facility, ICAR-Indian Agricultural Research Institute, New Delhi, India. Air temperature in the National Phytotron Facility was maintained as 22/18°C (±2°C) day/night temperature; 10/14 h light/dark photoperiod; 45% relative humidity and 450 μmol m^-1^s^-1^light intensity. The genotypes were screened for salt tolerance at seedling stage under hydroponic conditions as per nutrient composition suggested by Samineni *et al*. [34]. Seedlings were grown in above conditions for 4 d before the initiation of salt treatment. The nutrient solution was salinized by adding 40, 80 and 120 mM NaCl along with control without NaCl (but with 0.2mM Na^+^ from Na_2_SiO_3_ and 0.05mM Cl^−^ in the micronutrient stock). The nutrient solution was renewed every 3^rd^ d to maintain nutrient concentration. The duration of salt stress period was 30 d and each treatment was replicated thrice, with six plants in each treatment. At each sampling, plants were separated into roots and shoots; roots were washed three times in distilled water to remove the external solution. Surface water was blotted off using paper towels and fresh mass was measured. Tissues were oven dried at 65°C for 72 h and the dry mass was determined. After 30 d of saline treatment, data were recorded on seedling survival, growth (root and shoot length) and biomass (fresh and dry weight).

All the genotypes were grouped into three categories according to their response to salinity on plant survival under 120 mM NaCl as (i) salt-tolerant: ≥92% seedling survival; (ii) moderately tolerant: ≥40% seedling survival (and (iii) salt-sensitive: no seedling survival. After 30 d of salt stress treatments, salt tolerance was determined for the seedling survivability and salinity scores. Seedling survivability was calculated as follows: Seedling survivability % = ratio of seedlings survived the salinity stress to the total number of seedlings used x 100. **S**alinity tolerance was estimated on the basis of visual detection of salt injury following 1–5 score scale: 1 = healthy plants with no visible symptoms of salinity stress, 2 = green plants with slight yellowing of leaves, 3 = green plants with yellowing of leaves and necrosis of the margins of older leaves, 4 = necrosis of leaves with green stem, 5 = partial and completely dried leaves and/or stem.

Most tolerant breeding lines (PDL-1 and PSL-9), most tolerant wild accessions (ILWL-9, ILWL-137, ILWL-95, ILWL-203 and ILWL-428) and most sensitive cultigens (L-4147 and L-4076) were further selected for analysis of their physio-anatomical components after 10 days in 120 mM NaCl stress. Fifteen seedlings in triplicate were evaluated for determination of physiological and biochemical traits for salt tolerance. The growing conditions for the physiological analysis were similar to the seedling experiments for growth parameters. The following procedure were followed for various analysis:

#### Mineral analysis (Na^+^_,_ K^+^ and Cl^-^)

For mineral analysis, dry material of roots and shoots was ground in a pestle and mortar and digested in a mixture of sulphuric acid: nitric acid: Perchloric acid(H_2_SO_4_, HNO_3_ and HClO_4_) acids (1:10:4). Na^+^ and K^+^ were estimated on dry weight basis by the Flame Photometer (Systronics 128, India). Tissue chlorides were measured by chloridometer (Buchler Digital Chloridometer).

#### Detection of H_2_O_2_

For detection of H_2_O_2_, fifteen salinity treated root tips were collected from each treatment, excised and placed into a solution containing 200 mM calcium chloride (CaCl_2_) (pH 4.4) and 10 mM FDA (Fluorescein diacetate) for 15 min. The FDA fluorescence was detected under a fluorescence microscope according to Hempel *et al*. [35].

#### Determination of proline

Proline was determined by following the method of Bates *et al*. [36]. It was extracted from 0.5 g fresh leaf in 3% (w/v) aqueous sulphosalycylic acid and the homogenate was filtered. After addition of 2 ml ninhydrin and 2 ml glacial acetic acid, mixture was heated at 100°C for 1 h in water bath and then extracted with toluene (4 ml). For measurement of proline concentrations, absorbance was measured at 520 nm. Proline concentration was determined as μmol g^-1^FrWt^-1^ using a standard curve.

#### Determination of antioxidant enzymes

Six shoot samples from each treatment were collected for determination of antioxidant enzyme activities after 10 d exposure to salinity stress. The SOD, APX, GPX and CAT were extracted by grinding 1.0 g fresh shoot and root samples in 10 ml extraction buffer (0.1 M phosphate buffer, pH 7.5, containing 0.5 mM ethylene diamine tetra acetate EDTA in case of SOD, CAT and GPX and 1 mM ascorbic acid for APX). The extract was passed through four layers of cheese cloth and then centrifuged. Thereafter supernatant was collected and used as enzyme extract. Assays for SOD, APX, GPX and CAT were conducted following the methods of Aebi, Dhindsa *et al*., Nakano and Asada; Castillo *et al*., respectively [37–40].

#### Root and shoot anatomy

For studying root anatomy under control and salinity stress (120 mM NaCl), method outlined by Krishnamurthy *et al*., with slight modification was used. Free hand cross sectioning, not more than 50μm of stem (1 cm of the first internodes) and root (1 cm from the root tip) was done with a razor blade and stained with 50% toludine blue (polychromatic stain) [41]. Five plants per replication for tolerant and sensitive genotypes were used and uniform sections were observed for comparison. Observations and photography were done under optical microscope at 10x (Zeiss, AXIOSKOP 2).

### Evaluation of genotypes under artificially created saline soil

Based on the overall ranking of the genotypes for salinity tolerance at the seedling stage, two salt tolerant breeding lines and two salt sensitive cultigens were selected for characterization of tolerance during plant ontogeny under saline field conditions. The experiments were carried out on the saline plots under All India Co-ordinated Research Project on Salt affected Soil and Saline Use in Irrigation Water at RBS College, Bichpuri Agra, India during 2013–14 and 2014–15. The field plots (4.0 m x4.0 m) were lined by polythene sheets down to a depth of 0.9 m to avoid lateral influx of salts and water. The irrigation water was prepared synthetically by adding the salts viz., SARiw 10 (mmol)^1/2^, Ca/Mg ratio 1:1.6 and Cl:SO_4_ ratio 4:1. The electrical conductivity (EC) of the irrigation water was 4.0, 8.0 and 12.0 dSm^-1^. The electrical conductivity of the best available normal irrigation water ranged from 0.3 to 0.5 dS m^-1^. The crop was grown in fixed plots irrigated with different saline water treatments. The different EC salt solutions were added to soil at vegetative and flowering stages. Five plants were sampled at 40 and 90 d after salinization for analysis of Na^+^, Cl^-^ and K^+^ concentrations. The data were also recorded for number of pods/plant and seed yield/plant. The average minimum and maximum temperature during the experiment was between 11.3 to 27.1°C and 12.5 to 23.3°C during 2013–14 and 2014–15. The amount of total rainfall received was 20.8 mm and 34.8 mm during 2013–14 and 2014–15, respectively. Each experimental plot had 6 rows of 5 meter length with inter and intra row sowing 20 cm and 2.5 cm, respectively. Experiments conducted in triplicate in randomized block design and average values were used for interpretation. Non saline area was irrigated with water having 5 cm ECiw. Normal packages and practices were followed. Soil samples were collected at sowing and after harvest at 0–15 cm and 15–30 cm depth and dried to pass through 2mm sieve and analysed for pHs, ECe and SARe of saturation extract as per procedure of Richards [42].The filteration rate was determined by using ring method. Electrical conductivity (EC), pH, and SAR were determined in a saturated paste extract (Table 2).

**Table 2:** Determination of soil pH, ECe (dS/m), and SARe (mmole/l)^1/2^ at sowing and harvesting.

Treatment	Soil depth (cm)	2013–14						2014–15					
		At Sowing			At harvest			At sowing			At harvest		
		ECe (dS/m)	pHs	SARe (mmol/l)^1/2^	ECe (dS/m)	pHs	SARe (mmol/l)^1/2^	ECe (dS/m)	pHs	SARe (mmol/l)^1/2^	ECe (dS/m)	pHs	SARe (mmol/l)^1/2^
Control	0–15	1.7	8.5	5.6	2.2	8.5	5.8	1.8	8.4	5.8	2.3	8.5	6.2
	15–30	1.8	8.6	5.4	2.0	8.5	5.7	1.9	8.5	5.5	2.0	8.4	5.9
ECiw 4	0–15	2.3	8.5	6.7	3.3	8.4	8.6	2.4	8.4	6.9	3.5	8.4	9.1
	15–30	2.2	8.5	6.4	2.8	8.4	7.6	2.4	8.5	6.4	3.1	8.3	8.9
ECiw 8	0–15	4.1	8.4	7.6	7.2	8.4	10.2	4.3	8.4	7.9	8.0	8.4	12.8
	15–30	3.7	8.5	6.8	6.5	8.5	9.3	4.0	8.5	7.1	7.4	8.4	11.9
ECiw 12	0–15	5.9	8.4	8.1	11.8	8.3	14.3	6.2	8.4	11.9	12.3	8.3	15.3
	15–30	4.8	8.6	7.6	10.2	8.4	12.3	5.1	8.4	10.9	10.8	8.3	13.5

SARiw- Sodium Adsorption Ratio of irrigation water; SARe- Sodium Adsorption Ratio of soil saturation paste; ECiw- Electrical conductivity of irrigation water and ECe- Electrical conductivity of soil saturation paste

### DNA extraction

Leaves from 10 plants per genotype were used for extraction of genomic DNA. CTAB (Cetyl trimethylammonium bromide) method described by Doyle and Doyle with some modifications was used for extraction [42]. Ultra high resolution agarose (1%) was used to estimate the quantity of genomic DNA (deoxyribonucleic acid) against the lambda uncut DNA and quality was confirmed by spectrophotometer. DNA sample of 50 ng/μl was used as standard working concentration.

### SSR marker analysis

Out of different SSRs reported by Hamwieh *et al*., Kaur *et al*. and Jain *et al*., 495 SSR primers were used for genetic diversity study which had GC content greater than 35 [28, 43, 44]. Out of these primers, 30 were found to be polymorphic between salinity tolerant breeding lines (PDL-1 and PSL-9) and salt sensitive cultigens (L-4076 and L-4147). These selected primers were used for molecular assortment of 162 genotypes. The primers were synthesized by Microgen, South Korea and IDT, USA. Polymerase chain reaction (PCR) was performed in 10 μl reaction mixture, comprising 1 X PCR buffer, 0.1 U *Taq* DNA polymerase, 1 μM dNTP, 0.5 μM of forward and reverse primers each (Microgen, South Korea and IDT, USA) and 50 ng/μl of genomic DNA in a thermocycler (Agilent Technologies, USA). The PCR protocol was comprised in initial denaturation step of 94°C for 3 min followed by 40 cycles of 94°C for 30 sec, annealing at 55°C for 30 sec, elongation at 72°C for 1 min with a final extension at 72°C for 10 min. The amplified products were resolved on 3% ultra high resolution agarose gels and documented using Syngene Gel Doc System.

### Genetic diversity analysis

The 30 SSR markers were used for genetic profiling of 162 genotypes and scored on the basis of difference in allele size. The genetic distance, polymorphism information content (PIC) and major allele frequency based clustering was performed with NJ tree using Power Marker v3.25 software [45] and the MEGA 4.0 software was used to construct the dendrogram [46] following the bootstrap analysis with 1000 permutations for all the genotypes using Mega 4.0 software. The population structure for 162 wild and cultivars genotypes was deduced using Structure 2.3.4 software [47]. Further, Structure Harvester was used for the result analysis and construction of Evanno plots [48, 49]. The admixed model with independent allele frequency and a uniform prior probability of the number of populations, *K* was used in structure. The software was set for *K* = 1 to 15 with 10,000 MCMC replicates after a burn-in of 1, 00,000 replicates. Seven independent runs for each value of K were done to generate an approximate number of sub-populations [47]. The relation between genetic similarity identified by SSR markers and taxonomic distance measured by mean genetic distance and total sodium percentage were analysed using Jaccard’s Similarity Index and average taxonomic distance was calculated by NTSYS-pc v2.1 software [50].

### Statistical analysis

Duncan’s Multiple Range Test (DMRT) (*P* = 0.05) was used to evaluate differences among clusters for significance by using SAS 9.4 software. Data for morpho-physiological were analysed using two-way ANOVA to determine if significant differences were present among means. Variances were checked by plotting residual vs. fitted values to confirm the homogeneity of the data. Differences among the mean values were assessed by Least Significant Differences (LSD). Relationships between individual variables were examined using simple correlations which were also performed using SAS 9.4 software. Spearman’s rank correlation test (rs) was used to examine consistency in the rankings of genotypes for salt tolerance and seed yield between the hydroponic and field experiments.

## Results

### Morpho-anatomical components of salt tolerance under hydroponics

**Fig 1:**
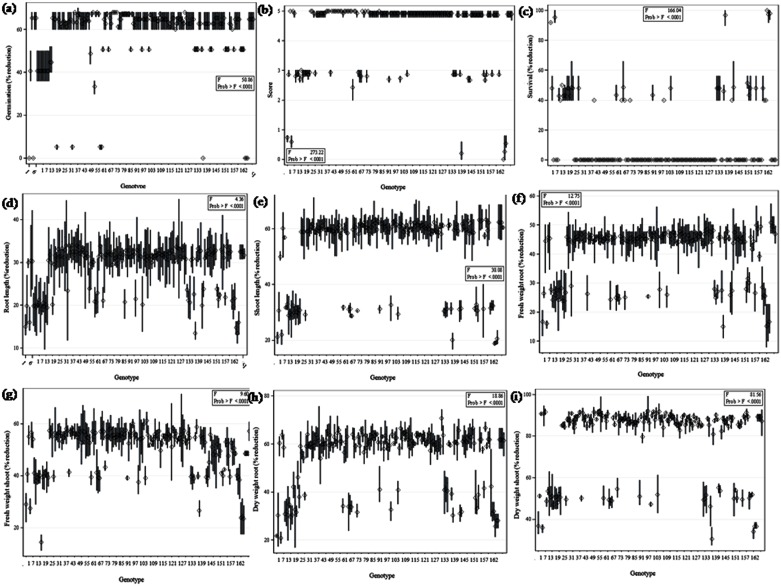
Seed germination percentage (a), salinity tolerance score (b), seedling survival percentage (c), root length (d), shoot length (e), root fresh weight (f), shoot fresh weight (g), root dry weight (h) and shoot dry weight of lentil genotypes raised (i) under 120 mM NaCl, in hydroponic conditions. Bar represents dispersion among observations and square represents mean value.

A set of 162 genotypes were assessed for salinity tolerance using germination, visual salt injury (scores), seedling survivability, reduction in root and shoot length, fresh and dry weight of roots and shoots. At 120 mM concentration all the genotypes showed delayed in seed germination and greatest variability in salt stress tolerance. At this level of saline stress, no reduction in seed germination occurred in tolerant wild accessions (ILWL-9, ILWL-95, ILWL-137, ILWL-418, ILWL-428 and ILWL-203) and breeding lines (PDL-1 and PSL-9), while sensitive genotypes showed 60 to 70 per cent reductions (Fig 1a). Visual symptoms of salt injury were first observed in the oldest leaves and then progressed successively towards younger ones (from base to upwards). Initial symptoms appeared in 120 mM NaCl treatment after 5 d, then 10 d later in the 80 mM treatment, and another 25 d later in the 40 mM treatment. The sensitive cultigens (L-4076 and L-4147) showed maximum salt injury with score of 5.0, whereas tolerant breeding lines (PDL-1 and PSL-9) and tolerant wild accessions (ILWL-9, ILWL-95, ILWL-137 and ILWL-428) exhibited minimum injury with score of 0–1.0 at 120 mM NaCl (Fig 1b). After two weeks, the leaves of moderately tolerant genotypes (L-4594, PDL-2, L-7903, IG-109039, IG-136607 and L-4590) in 120 mM NaCl started showing necrotic spots which spread slightly along with wilting. Leaves of sensitive cultigens (L-4147 and L-4076) and landrace (IG-9) dried up and plants began to die in 15d (S1 Fig). Tolerant breeding lines and wild accessions (PDL-1, PSL-9 and ILWL-09, ILWL-137, ILWL-96 and ILWL-428) exhibited ≥90%seedling survival at 120 mM NaCl and were classified as salt-tolerant. Some genotypes showed ≥40% seedling surviving at 120 mM NaCl and were classified as moderately salt tolerant. Rest of the genotypes were classified as sensitive showing no survival at 120 mM NaCl. The genotypes showed less difference in seedling growth and biomass at lower than at higher salinity in all the genotypes. The seedling growths (root and shoot length) and biomass (fresh and dry weight of roots and shoots) decreased markedly in all the genotypes at 120mM NaCl. However, the reduction in root and shoot length, fresh and dry weight of roots and shoots was lower in tolerant wild accessions and breeding lines as compared to moderately tolerant and sensitive genotypes (Fig 1d–1i).

**Fig 2:**
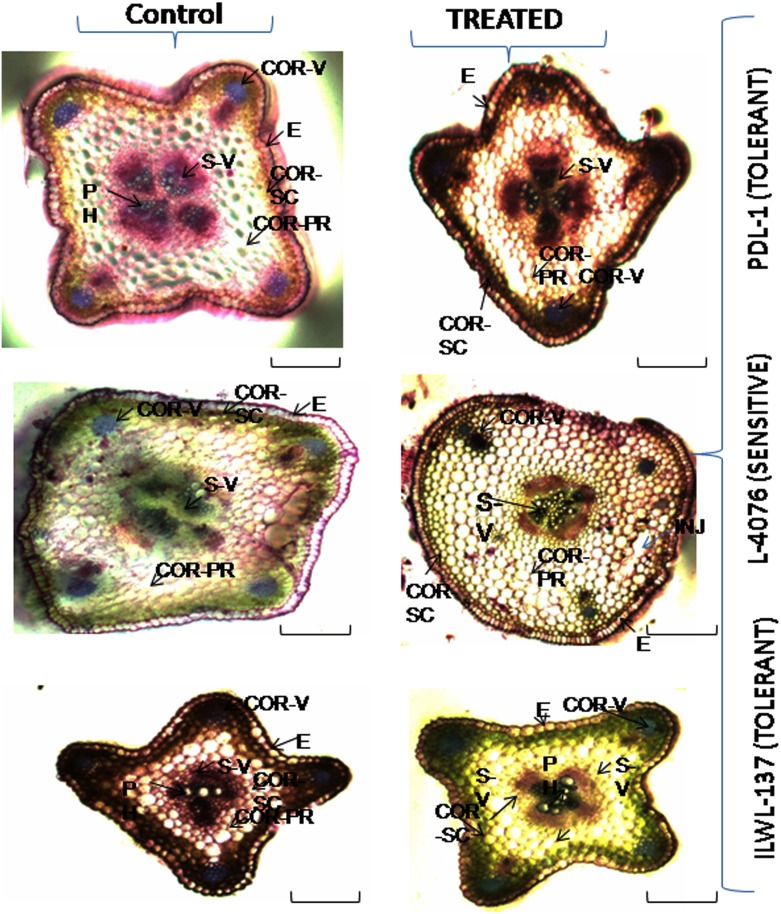
Changes in stellar region and vascular bundles in cross-sections of stem in tolerant and sensitive *Lens* species raised under control (0 mM) and (120 mM NaCl) saline stress in hydroponics. E-Epidermis, COR-SC—Cortical-Schlerenchyma, COR-PR- cortical parenchyma, PH = Pholem, END = Endodermis, S-V = Stellar-Vascular bundle, COR-V = Cortical -Vascular bundle. Bar in each figure represents 100 μm.

**Fig 3:**
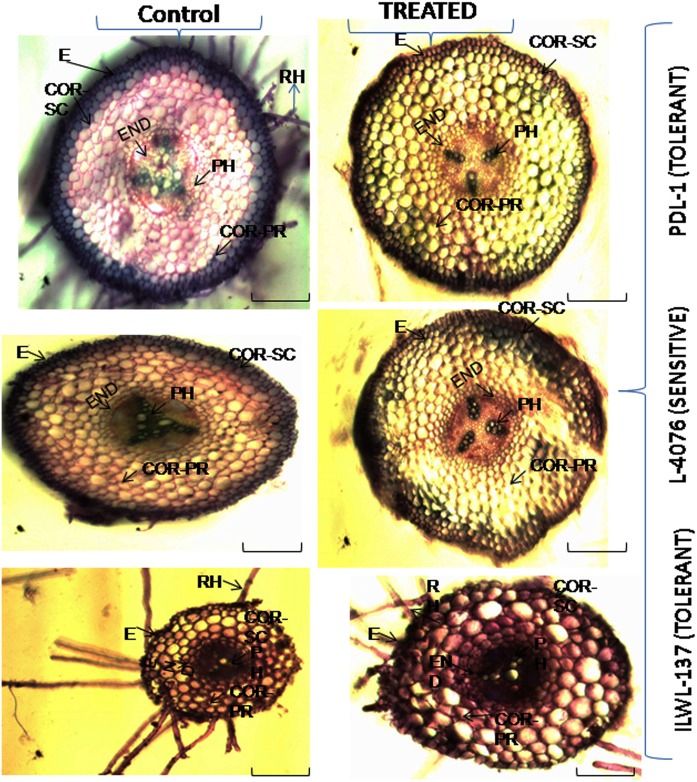
Changes in stellar region and vascular bundles in Root cross-sections of tolerant and sensitive *Lens* species raised under control (0 mM) and (120 mM NaCl) saline stress conditions raised in hydrophonics. E = Epidermis, COR- SC = Cortical-Schlerenchyma, COR- PR = Cortical Parenchyma, PH = Phloem, RH = Root Hair. Bar in each figure represents 100 μm.

Overall observations on anatomical features under control for stem and root depicted that tolerant wild accession (ILWL-137) and breeding line (PDL-1) contained similar anatomical structures, though their vascular arrangement in the stele region varied (Fig 2). The root and stem cross-sections of tolerant wild accession were smaller than sensitive cultigen (L-4076). In stem, the epidermal cells of tolerant breeding lines and wild accession were larger than that of sensitive cultigens. All the genotypes had two types of vascular systems, one at the stellar region and four patches in the cortical region. Vascular systems were deeply stained and well organised in the tolerant breeding line and wild accession when compared to sensitive cultigens, where the vascular systems were less stained and disorganised. The overall anatomical structure of tolerant breeding line and wild accession was a butterfly shape, whereas sensitive cultigen had square type under control condition (Fig 2). Root of tolerant breeding line had thicker epidermis along with three layers of sclerenchymatous cortex and prominent endodermis and pericycle layer in comparison to sensitive cultigens. The roots of tolerant wild accessions and breeding line and sensitive cultigen were similar, having 8–9 cortical rings from stele and organised stellar region, though sensitive cultigen had larger cortical cells than that of tolerant breeding line. Tolerant wild accession with small cross-sectional area had 6–7 cortical rings where large cortical cells observed. Root hairs were more prominent in tolerant breeding lines and wild accession than sensitive ones (Fig 3).

Under salinity stress, wild genotypes were least affected. In case of stem, epidermis rupturing and cortical cell enlargement with rupturing at certain region were also noticed in sensitive cultigen, whereas in tolerant breeding line epidermal layer was found intact and showed no damage. There was no hypodermal deposition in sensitive cultigen with highly shrunken stellar area in comparison to its control. Cortical vascular bundles were found intact in tolerant breeding line and wild accession, whereas in sensitive cultigen, it was shrunken. Tolerant wild accession showed similar response to that of tolerant breeding lines. In case of root, tolerant breeding line deposition was restricted to upper layers, whereas in case of sensitive cultigen it was found in patches within many layers towards the stele along with cortical cell rupturing at certain regions. Shrunken stele area along with distorted phloem vessels were visualised in sensitive cultigen. There were observed very less changes in stellar region in tolerant breeding line and tolerant wild accession as compared to control (Fig 3).

### Physiological components of salt tolerance under hydroponics

**Fig 4:**
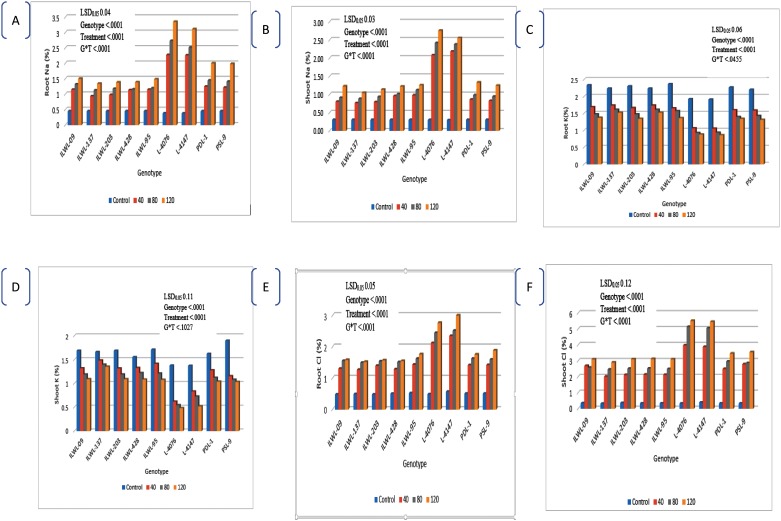
Na^+^, K^+^, Cl^-^ contents in root and shoot of Lens species at different levels of salt stress (0, 40, 80 and 120mM NaCl) at seedling stage under hydroponic conditions.

The distribution of Na^+^, Cl^−^ and K^+^ in roots and shoots was significantly affected at seedling stage under 120 mM NaCl concentration. The concentrations of Na^+^ and Cl^-^ increased in roots and shoots of all the genotypes, but the increase was less in tolerant wild accessions (ILWL-09, ILWL-95, ILWL-317, ILWL-203, and ILWL-428,) and tolerant breeding lines (PDL-1 and PSL-9) than sensitive cultigens (L-4076 and L-4147) under all the treatments (Fig 4a–4f). K^+^ concentration decreased in roots and shoots of all the genotypes after treatment with 120- mM NaCl. However, uptake of K^+^ in sensitive cultigens was less as compared to tolerant breeding line and wild accession (Fig 4c and 4d). The sensitive and tolerant genotypes had almost similar Na^+^ and Cl^-^ concentrations in roots and shoots under normal condition. K^+^ concentration of plants was lower in the sensitive than in the tolerant genotypes under same conditions.

**Fig 5:**
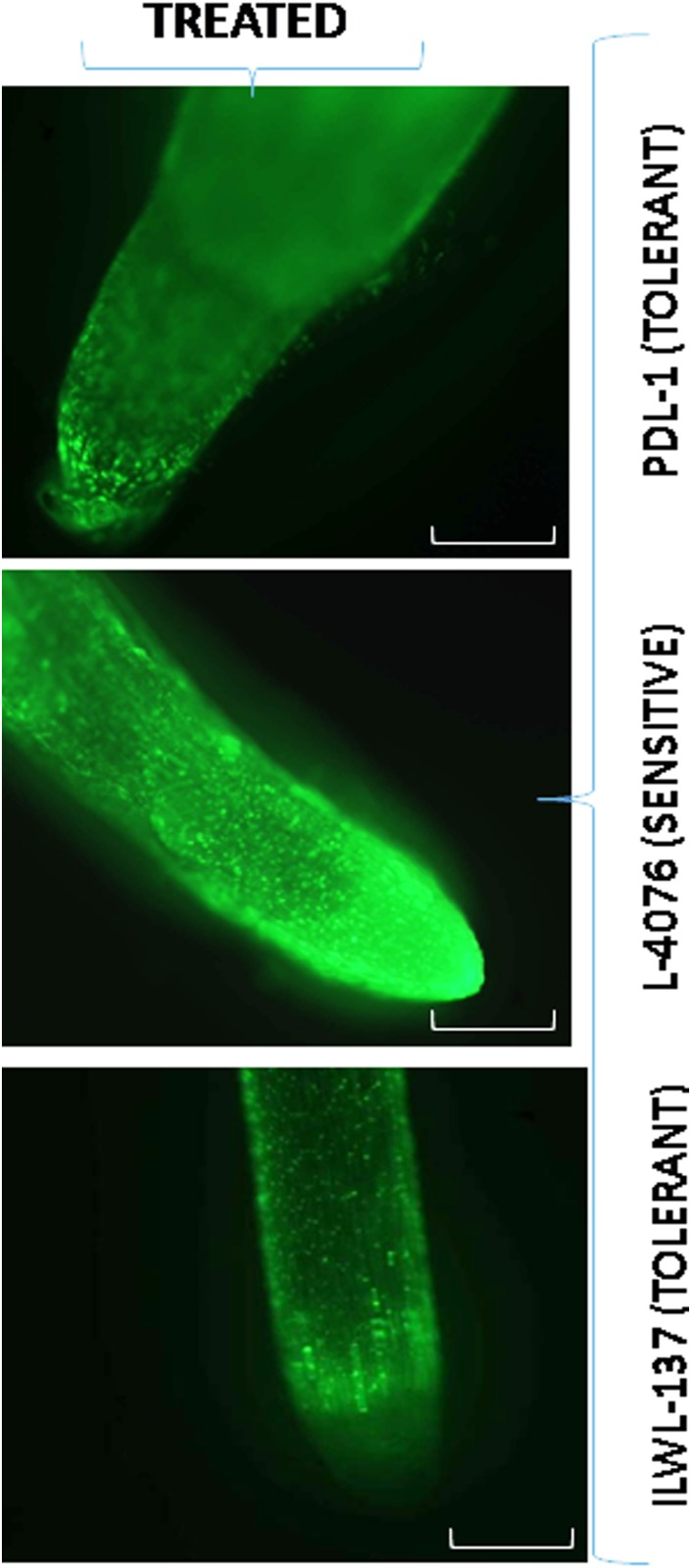
Fluorescent root images of tolerant breeding line (PDL-1), sensitive cultigen (L-4076) and tolerant wild accession (ILWL-15) for detection of H_2_O_2_ under 120 mM NaCl stress conditions in hydrophonics. Bar in each figure represents 1 mm.

For ROS detection under salt stress, the roots were stained with FDA fluorescent, which showed the presence of H_2_O_2_. FDA fluorescence was negligible without salt stress (fig not shown), and markedly increased at 120 mM NaCl. A low fluorescent was observed in tolerant breeding line (PDL-1) and wild accession (ILWL-137 as compared to sensitive cultigens (L-4076) (Fig 5).

**Fig 6:**
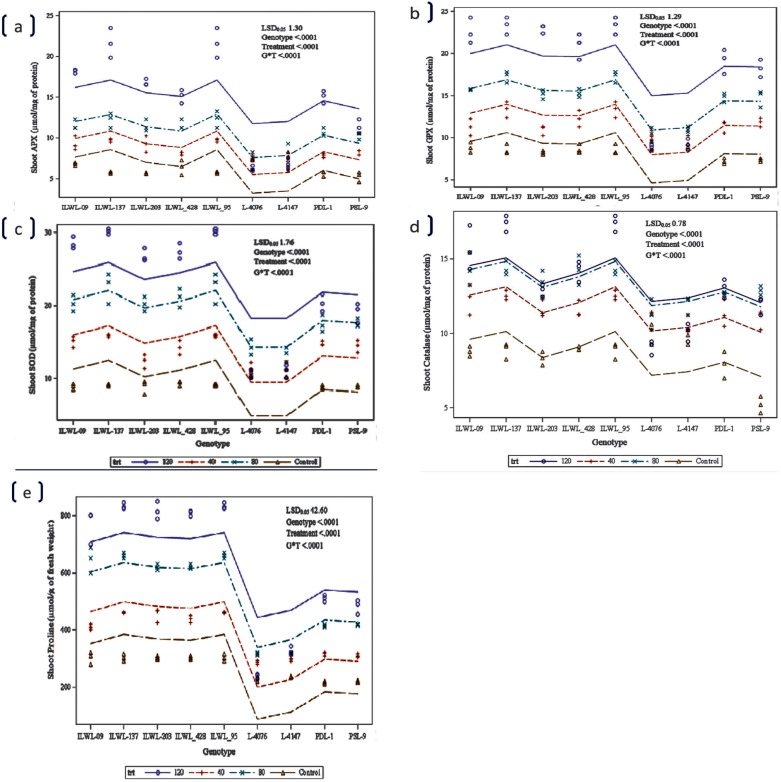
Effects of salt stress (120mM NaCl) on activity of antioxidant enzymes APX (a), GPX (b), SOD (c), catalase (d) and proline concentration (e) in root and shoot of *Lens* spices raised in hydrophonics.

The activity of SOD, APX, GPX and CAT enzymes in shoots was higher under salt stress than control in all the genotypes. The extent of increase was significant in the tolerant wild accessions and breeding lines than sensitive cultigens (Fig 6a–6c). However, the activity of CAT enzyme did not show any significant increase in sensitive cultigens (Fig 6d). During salinity stress, significant increase in proline concentration was observed. The salt increased proline concentrations and it was always higher in the tolerant wild accessions and breeding lines than at the sensitive cultigens in all the salinity levels (Fig 6e**)**.

### Response of yield attributes under saline field conditions

**Fig 7:**
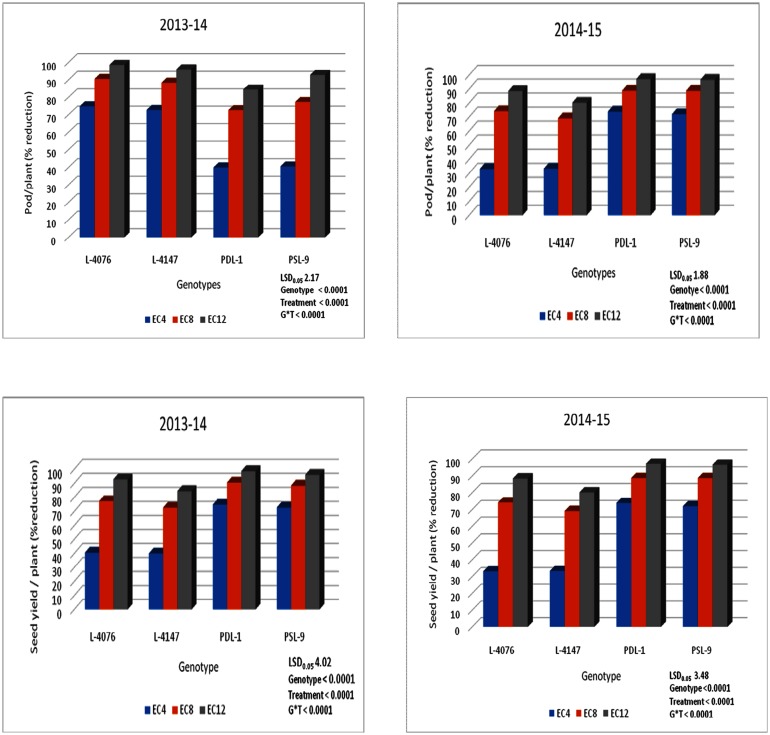
Percent reduction in pods and seed yield per plant in contrasting lentil genotypes grown under three salt concentrations during 2013–14 and 2014–15.

**Fig 8:**
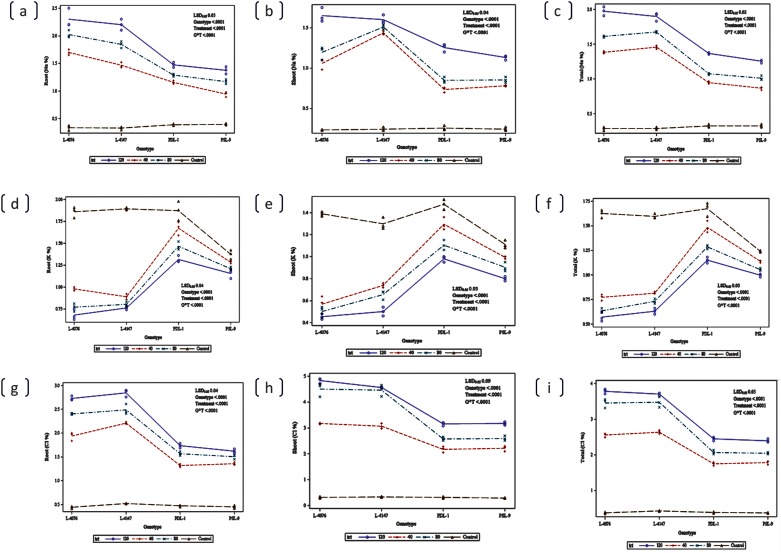
Changes in Na^+^ (a and b), K^+^ (c and d) and Cl^-^ (e and f) content in root and shoot of contrasting lentil genotypes at vegetative stage in control (EC<1.0) and saline stresses (40, 80 and 120 ECiw) under field conditions. Circles are the actual observations and line represents mean value.

**Fig 9:**
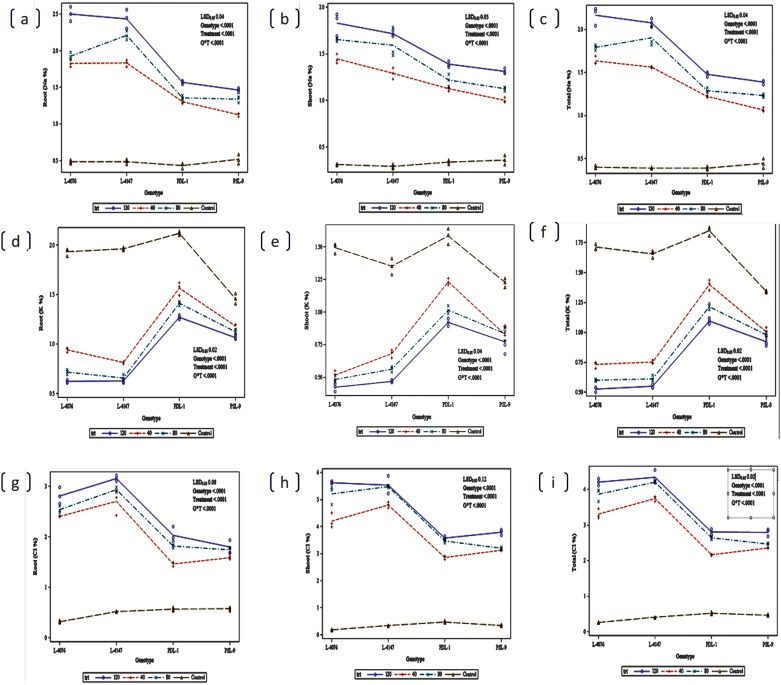
Changes in Na^+^ (a and b), K^+^ (c and d) and Cl^-^ (e and f) content in root and shoot of contrasting lentil genotypes at reproductive stage in control (EC<1.0) and saline stresses (40, 80 and 120 ECiw) under field conditions. Circles are the actual observations and line represents mean value.

Genotypes responded differently to different salinity levels and the reduction in seed yield varied between 45.5 to 86.5% at 4 ECiw, 76.5 to 88.8% at 8 ECiw, 88.5 to 97.1% at 12.0 ECiw of saline irrigation water during 2013–14. However, the reduction in seed yield was lower during 2014–15. Tolerant breeding lines (PDL-1 and PSL-9) exhibited minimum reduction in pod and seed yield/plant, whereas sensitive cultigens (L-4076 and L-4147) performed poorly as a result they showed maximum reduction in pods and seed yield/plant compared to the control (Fig 7a and 7b). Similar results were recorded during 2014–15 (Fig 7c and 7d). It is evident from above findings that these varieties had variable degree of salinity tolerance. Significant genotypic variation occurred at Na^+^, Cl^-^ and K^+^ concentration (Figs 8 and 9). The amount of Na^+^ in the roots was found higher than that in the shoots. However, uptake pattern of Cl^-^ was high in shoots as compared to roots. At 12.0 ECiw saline irrigation, roots and shoots accumulated maximum amount of Na^+^ and Cl^-^ in all the genotypes. However, accumulation was relatively lower in tolerant genotypes (Figs 8 and 9). When K^+^ content in roots and shoots was analysed, it declined drastically in tolerant and sensitive genotypes at 12 ECiw. The sensitive cultigens exhibited much smaller amount of K^+^ compared to tolerant breeding lines. The accumulation of Na^+^ and Cl^−^ was lower and K^+^ was higher at vegetative stage as compared to reproductive stage in all the genotypes (Figs 8 and 9).

### Correlation between hydroponic and field screening techniques

Significant correlation was recorded between the two screening techniques for morpho-physiological traits, such as reduction in germination per cent, root length, shoot length, fresh and dry weight of roots and shoots, seedling survival, salinity scores, total sodium, potassium and chloride and reductions in seed yield (Table 3). Based on the significant correlation among the traits in the field as well as hydroponic conditions it is concluded that hydroponic method is simple, time saving and easy for screening of large number of genotypes for salinity tolerance at the seedling stage.

**Table 3:** Pearson correlation coefficients among traits recorded under hydroponic and field conditions.

	Hydroponic screening technique												Field screening technique				
Hydroponic screening technique		GM	RL	SL	FWR	FWS	DWR	DWS	S.S	Score	T. Na^+^	T. K^+^	FTNa^+^	FTK^+^	FTCl^-^	FRSY1314	FRSY1415
	GM																
	RL	0.807 **															
	SL	0.984 **	0.769 **														
	FWR	0.948 **	0.789 **	0.950 **													
	FWS	0.953 **	0.768 **	0.911 **	0.874 **												
	DWR	0.992 **	0.829 **	0.976 **	0.950 **	0.951 **											
	DWS	0.994 **	0.790 **	0.986 **	0.963 **	0.927 **	0.985 **										
	S.S	0.996 **	0.797 **	0.974 **	0.950 **	0.964 **	0.987 **	0.991 **									
	Score	-0.996 **	-0.800 **	-0.972 **	-0.950 **	-0.964 **	-0.989 **	-0.991 **	-0.999 **								
	T. Na^+^	-0.977 **	-0.744 **	-0.954 **	-0.914 **	-0.977 **	-0.979 **	-0.968 **	-0.984 **	0.985 **							
	T. K^+^	0.992 **	0.802 **	0.967 **	0.952 **	0.960 **	0.984 **	0.984 **	0.992 **	-0.995 **	-0.975 **						
Field screening technique	FTNa^+^	-0.976 **	-0.843 **	-0.953 **	-0.941 **	-0.962 **	-0.986 **	-0.972 **	-0.983 **	0.983 **	0.979 **	-0.970 **					
	FTK^+^	0.961**	0.769 **	0.954 **	0.914 **	0.900 **	0.954 **	0.960 **	0.954 **	-0.954 **	-0.942 **	0.953**	-0.923 **				
	FTCl^-^	-0.979 **	-0.761 **	-0.960 **	-0.957 **	-0.937 **	-0.968 **	-0.984 **	-0.988 **	0.983 **	0.969 **	-0.970**	0.968**	-0.953 **			
	FRSY1314	0.775 **	0.489 **	0.763 **	0.727 **	0.799 **	0.779 **	0.779 **	0.794 **	-0.797 **	-0.824 **	0.774**	-0.816**	0.615**	-0.763 **		
	FRSY1415	0.864 **	0.701 **	0.861 **	0.867 **	0.786 **	0.870 **	0.883 **	0.859 **	-0.865 **	-0.840 **	0.844**	-0.882**	0.735**	-0.842**	0.883 **	

GM = germination; RL = Root length; SL = Shoot length S.S = Seedling survival; FWR = Fresh weight root; FWS = Fresh weight shoot; DWR = Dry weight root; DWR = Dry weight root; T. Na+ = Total sodium; T. K^+^ = Total potassium; FTNa^+^ = Field total sodium; FTK^+^ = Field total potassium; FTCl^-^ = Field total chloride; FRSY13-14 = Field reduction in seed yield 2013–2014; FRSY2014-15 = Field reduction in seed yield 2014–2015. P = < .001)

**, designates significance at 1 percent level.

### Ranking of genotypes for salt tolerance between hydroponic and field screenings

All the diverse set of 162 genotypes were ranked based on observations obtained from hydroponic and field conditions during 2013–14 and 2014–15, to evaluate the consistency in their performance. The ranking of genotypes based on seedling survivability at 120 mM NaCl and reduction of seed yield in the field was found significantly correlated (r = 0.883; P = 0.0001). Tolerant and sensitive genotypes showed similar response under both hydroponic as well as field conditions.

### Genomic scanning through SSR markers

A set of 495 primers were pre-screened in most salt tolerant breeding lines (PSL-9, PDL-1) and sensitive ones (L-4147 and L-4076), of which 30 SSR primers exhibiting polymorphism, were selected for genetic diversity analysis among 162 genotypes (Table 4). All these 30 SSR primer pairs generated polymorphic bands among the genotypes (Figure not shown). A total of 137 alleles were identified with an average of 4.57 alleles per locus. The number of alleles per locus ranged from 3 (PLC_39, PBA_LC_376, PBA_LC_1308, PBA_LC_404, PBA_LC_1241) to 8 (PBA_LC_1401, LC_02). The major allele frequency varied between 0.253 (LC_02) to 0.788 (LC_39 and LC_104), respectively with a mean value of 0.557. The gene diversity and PIC values varied between 0.356 and 0.809 and 0.321 and 0.782, with an average of 0.571 and 0.515, respectively. The primer which showed highest gene diversity and PIC values was LC_02, while the one with lowest gene diversity and PIC values were the primers PLC_39 and PLC_81. Heterozygosity in all the genotypes ranged from 0 to 0.3 with a mean value of 0.053 and the highest heterozygosity was observed in PBA_LC_1400 (Table 4) (S1 Table).

**Table 4:** Allelic variation and PIC values for 30 SSR identified in 162 lentil genotypes.

Marker	Major Allele Frequency	Allele No	Gene Diversity	Heterozygosity	PIC
LC-01	0.6563	6	0.5175	0.1688	0.4716
LC-02	0.2531	8	0.8085	0.3000	0.7816
LC-16	0.4813	5	0.5722	0.0875	0.4795
PBA-LC-117	0.7688	5	0.3844	0.0313	0.3551
PBA-LC-118	0.4813	5	0.6447	0.0188	0.5797
PBA-LC-1241	0.4500	3	0.6084	0.0000	0.5254
PBA-LC-1247	0.6063	4	0.5563	0.0000	0.4979
PBA-LC-1308	0.6313	3	0.5193	0.0000	0.4523
PBA-LC-1363	0.3813	5	0.6955	0.0438	0.6421
PBA-LC-1401	0.5625	8	0.6316	0.2625	0.5971
PBA-LC-1698	0.6063	4	0.5596	0.0125	0.5034
PBA-LC-216	0.4750	4	0.6364	0.0063	0.5679
PBA-LC-221	0.6000	4	0.5853	0.0625	0.5440
PBA-LC-222	0.4188	4	0.6440	0.0125	0.5754
PBA-LC-368	0.4188	5	0.6583	0.0313	0.5917
PBA-LC-376	0.6313	3	0.5007	0.0000	0.4197
PBA-LC-377	0.5625	4	0.6103	0.0063	0.5613
PBA-LC-379	0.5438	5	0.5769	0.1000	0.5004
PBA-LC-383	0.6938	4	0.4677	0.0375	0.4182
PBA-LC-404	0.5125	3	0.5616	0.0000	0.4673
PBA-LC-652	0.6250	4	0.5494	0.0438	0.5011
PLC-05	0.3938	5	0.6759	0.1250	0.6182
PLC-100	0.2906	6	0.7755	0.0313	0.7387
PLC-104	0.7875	6	0.3651	0.0063	0.3468
PLC-30	0.5375	4	0.6096	0.0250	0.5458
PLC-35	0.5688	5	0.6026	0.1188	0.5532
PLC-39	0.7875	3	0.3560	0.0000	0.3262
PLC-51	0.5813	4	0.5805	0.0000	0.5223
PLC-81	0.7813	4	0.3586	0.0188	0.3206
PLC-91	0.6281	4	0.5172	0.0438	0.4469
Mean	0.5572	4.6	0.5710	0.0531	0.5151

H, Heterozygosity; PIC, Polymorphism information content.

### Cluster analysis of DNA polymorphism

**Fig 10:**
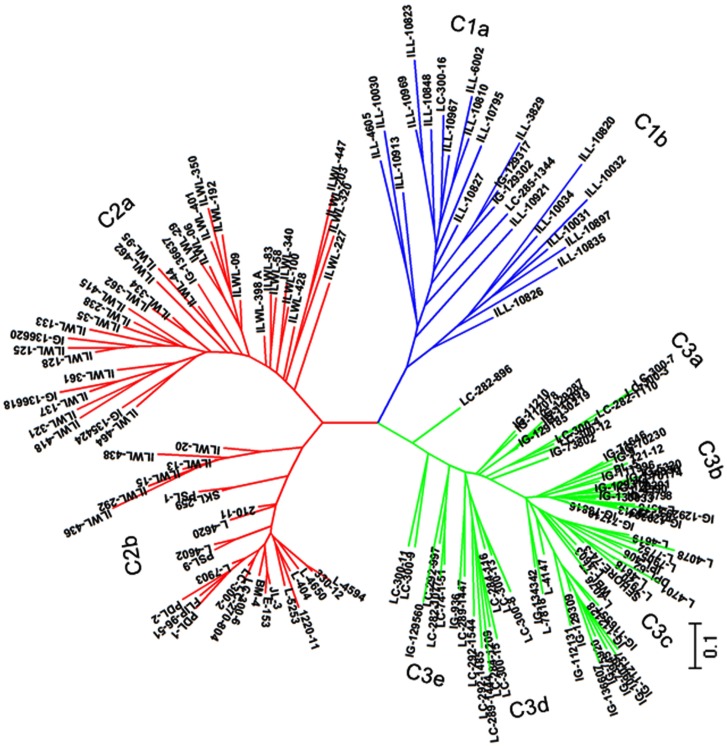
UPGMA tree based on dissimilarity index of 30 SSR markers for 162 lentil genotypes.

The genetic relationship among lentil genotypes has been represented in unweighted neighbour joining (UNJ) dendrogram constructed on the basis of informative SSR alleles (Fig 10) which grouped the genotypes into 3 major clusters. Cluster 1 comprised of ILL-series of germplasm collections originating from ICARDA, which was further subdivided into two sub-clusters i.e. C1a and C1b. Cluster 2 was subdivided into two sub-clusters, C2a having most of the wild genotypes and C2b most of the breeding lines, originating from India. Cluster 3 comprised of most of the remaining cultigens and divided into 5 sub-clusters, where sub-clusters C3d and C3e mostly comprised of LC series of breeding lines originating from India. Two genotypes (ILWL-227 and ILWL-203) were found to be quite distinct. The two tolerant breeding lines (PDL-1 and PSL-9) were grouped together in cluster 2b and the two sensitive cultigens (L-4147 and L-4076) grouped in cluster 3b.

### Population structure and genetic relationship

**Fig 11:**
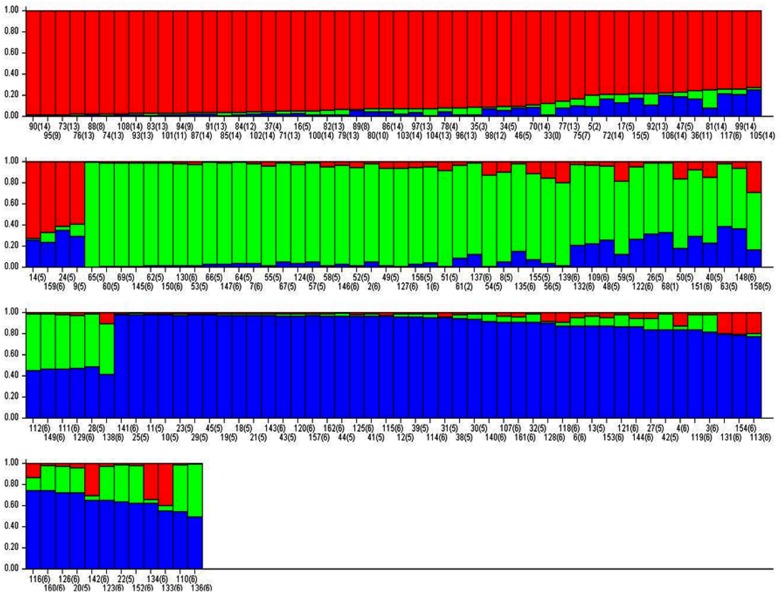
Structure plot with K = 3 depicting model based population, using structure with 30 SSR markers. The number in the bracket represents origin of accessions as follows: Argentina (1), Bangladesh (2), Crotia (3), France (4), ICARDA (5), India (6), Italy (7), Jordan (8), Lebnon (9), Palestine (10), Slovania (11), Spain (12), Syria (13) and Turkey (14).

Pritchard’s structure of 162 *Lens* genotypes was assayed from SSR allelic diversity data, where best goodness of fit was found at k = 3. Using a membership probability threshold of 0.80, 45 genotypes were assigned to SG 1 (red), 35 to SG 2 (green), 42 to SG 3 (blue) and 38 were found admixtures (AD). The population structure was mostly co-related to cluster data as the three populations were separated from wilds and ‘ILL’ series of cultigens. On the basis of origin, triangle plot depicted that the genotypes from ICARDA were distributed in all the population sub-groups. Those originating from India were mainly grouped into sub-groups SG 2 and SG 3. Most of the wilds originated from Syria, Turkey, France, Italy, Jordan, Palestine, Slovenia and Spain fell in group SG 1 (Fig 11)

### Cluster analysis for morpho-physiological parameters

**Fig 12:**
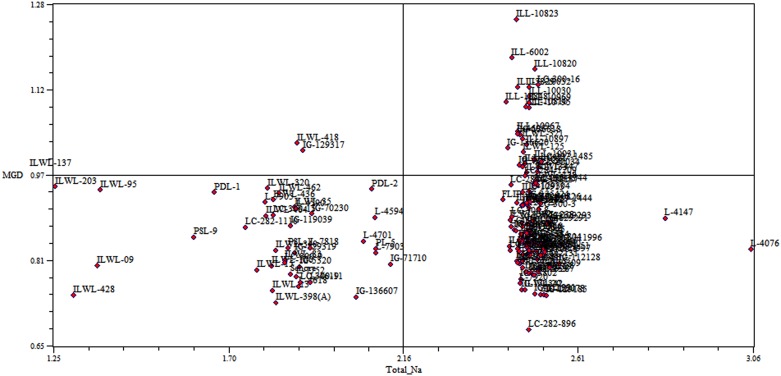
Correlation between genetic similarity index and taxonomic distance for total sodium per cent of 162 genotypes at 120 mM NaCl concentration.

Based on SSR markers, all the genotypes were characterized into seven clusters in respect of survival per cent, growth parameters, salinity score and total Na^+^ and K^+^ concentrations under salinity stress condition. Among the SSR clusters, there was found wide range in the values for most of the characters. Significant (*P* = 0.05) differences for all the characters were observed among the clusters. These parameters differed significantly among the genotypes of cluster 2.A which mostly comprised of wild accessions as compared to those of other clusters (Table 5). The lowest salinity score (3.9), reduction in germination (53.9), root length (27.8%), shoot length (49.6%), fresh root weight (38.3%) fresh shoot weight (46.5%), dry root weight (52.7%), dry shoot weight (73.6%), Na^+^ (2.2%) and K^+^ (0.88%) contents were observed in the tolerant breeding lines of cluster 2a (Table 5). These differences in the growth and physiological traits may be due to high salinity tolerance among tolerant wild accessions of cluster 2a. The clusters based on SSR markers have been found highly associated with the degree of salinity tolerance. The wild accessions with the similar degree of salinity tolerance were clustered into same groups. Most tolerant breeding lines were grouped in cluster 2b which showed greater proximity with cluster 2a in terms of genetic relatedness. Further, tolerant and sensitive genotypes were separated when correlation between genetic similarity index and taxonomic distance for total sodium percentage was evaluated using Jaccard similarity index under the salt stress conditions (120 mM NaCl) (Fig 12) (S2 Table).

**Table 5:** Cluster means of reductions in germination per cent (GM), root length (RL), shoot length (SL), seedling survival (S.S), fresh weight root (FWR), fresh weight shoot (FWS), dry weight root (DWR), dry weight root (DWR), total sodium (T. Na^+^ %) and total potassium (T.K^+^ %) under salt stress condition among the clusters.

Genotypes	GM%	RL	SL	FWR	FWS	DWR	DWS	survival	Score	T.Na^+^%	T. K^+^ %
CLUSTER 1a	60.8^BC^	31.0^ABCD^	58.7^CD^	44.6^BC^	54.2B^C^	60.3^CD^	85.6^D^	2.8^F^	4.8^C^	2.4^C^	0.79^G^
CLUSTER 1b	64.2^CD^	31.7^CD^	61.0^DE^	46.8^C^	53.3^ABC^	63.0^D^	88.1^E^	0.0^G^	4.9^B^	2.5^A^	0.78^HA^
CLUSTER 2a	53.9^A^	27.8^A^	49.6^A^	38.3^A^	46.5^A^	52.7^A^	73.6^A^	21.2^A^	3.9^H^	2.2^H^	0.88^A^
CLUSTER 2b	60.0^A^	28.1^AB^	50.2^A^	39.3^A^	48.9^AB^	51.6^A^	75.0^B^	17.7^B^	4.1^G^	2.3^G^	0.84^B^
CLUSTER 3a	62.6^C^	31.2^BD^	57.3^C^	44.2^BC^	54.4^BC^	58.3^CB^	85.8^D^	3.6^E^	4.8^D^	2.4^D^	0.79^E^
CLUSTER 3b	56.6^AB^	28.1^AB^	51.1^A^	39.0^A^	47.0^A^	53.1^A^	75.3^B^	14.7^C^	4.2^F^	2.3^F^	0.82^C^
CLUSTER 3c,d,e	57.8^AB^	29.5^ABC^	54.4^B^	42.2^B^	52.7^ABC^	56.5^B^	81.2^C^	8.5^D^	4.6^E^	2.4^E^	0.80^D^

Values within each column that do not share common letter are significantly different by Duncan’s post- hoc test at P_0.05

## Discussion

The morpho-physiological traits that underlie natural variation are largely unexploited and can be used for yield improvement. Such variation provides valuable information on the capacity and performance of different cultivars under different environmental conditions [51, 52]. The knowledge of variation in different cultivars can felicitate the development of salt tolerant varieties/commercial cultivars for cultivation in salt affected areas. The findings of present study clearly indicate that there is wide variation among the cultivars, breeding lines, landraces and wild accessions and can be exploited for development of salt tolerant varieties.

Seed germination and early seedling growth are considered major factors limiting crop growth under salt stress [53]. The results showed no reduction in germination of tolerant wild accessions and breeding lines at any salinity levels. However, moderately tolerant and sensitive cultigens exhibited decreased germination, when salinity was increased from 40 to 120 mM NaCl (Fig 1a). This might be due to of adverse effects of salt stress on water imbibition by seed and toxic effects of ions on the metabolism of seed during germination. These results are in agreement with Almodares *et al*. [54].

Visual symptoms of salt injury like yellowing of leaves, necrosis at leaf margins and ultimately drying of leaves and stems and seedling survival are early screening tools for salt-tolerance (Singh unpublished data). The tolerant wild accessions (ILWL-09, ILWL-95, ILWL-203, ILWL-137 and ILWL-428) and breeding lines (PDL-1 and PSL-9) showed partial salt injury symptoms with salinity score of 0.0–1.0 and sensitive ones exhibited higher salt injury with scores of 4.0–5.0 at 120 mM NaCl stress, respectively (Fig 1b). These symptoms could be the result of excess Na^+^ and Cl^-^ ions accumulation inducing chlorosis and leaf senescence as reported in other crops [55]. The chlorosis and leaf senescence may affect seedling survivability as a result of salinity stress.

Seedling survival in saline soil has been widely used parameter to evaluate the tolerance of crop plants. This criterion is potentially useful, because plant breeders may adopt it as the basis for selection of salt-tolerant lines and cultivars. Salinity stress at 120 mM NaCl levels greatly affected the seedling survival in tolerant, moderately tolerant and sensitive genotypes. Salinity of similar NaCl level in sensitive cultigens led to no seedling survival for 15 d. On the other hand, tolerant breeding lines and wild accessions continued to survive with more than 50 per cent seedlings, at similar salt stress (Fig 1c). This study evidenced that plant survival is crucial indicator for salt tolerance evaluation. The results are in concurrence with Murillo-Amador *et al* [56, 57].

Dry matter production is a potent indicator of plant’s performance under salt stress and is associated with seed yield production under saline conditions [58]. The seedling growth was found to be severely affected by salinity stress in various crop plants [5]. The seedling growth (root and shoot length) and biomass (fresh and dry weight of roots and shoots) accumulation in all the genotypes decreased with increase in salt stress. However, salt-sensitive cultigens showed more reduction in seedling growth and biomass as compared to tolerant and moderately tolerant genotypes (Fig 1d–1h). This suggests that the most sensitive cultigens (L-4076 and L-4147) failed to restrict to move Na^+^ and Cl^-^ ions from roots at 120 mM NaCl and thus plants could not survive under high salt stress and caused the drastic reduction in seedling growth. The tolerant breeding lines (PDL-1 and PSL-9) and wild accessions (ILWL-09, ILWL-95, ILWL-137, ILWL-203 and ILWL-428), exhibited better root and shoot growth and biomass accumulation with higher seedling survivability as compared to sensitive ones under high salt stress (120 mM). Among various parameters, seedling survivability could be ranked as first criterion for phenotyping of large population for salinity tolerance.

The effect of ions has been considered to be associated with salt-tolerance mechanism in crop plants [59, 60]. The Na^+^ and Cl^-^ ions are associated with salt tolerance with the ability to limit uptake and/or transport of ions from the roots to aerial parts. In the present study, the uptake of both Na^+^ and Cl^-^ showed a proportional increase with increase in salinity of the growth medium and highest uptake was observed at 120 mM NaCl. The uptake of Na^+^ and Cl^-^ was highest in sensitive cultigens compared to tolerant breeding lines and wild accessions (Fig 4a–4f). These variations in Na^+^ and Cl^-^ were probably due to influx rates. The Na^+^ concentration in shoot was lower than in roots, whereas it was reverse for Cl^-^ (Fig 4a, 4b, 4e and 4f). Thus, the present data indicated that Cl^-^ toxicity in shoots could be due to mechanisms causing salt sensitivity in *Lens* genotypes. These results are in agreement with those of Samineni *et al* [34]. Salt-sensitive cultigens (L-4076 and L-4147) increased Na^+^ and Cl^-^ concentrations in roots and shoots. These genotypes could be considered as accumulator of Na^+^ and Cl^-^ ions. Na^+^ and Cl^-^ concentrations were lower in roots and shoots of salt-tolerant wild accessions (ILWL-9, ILWL-95, ILWL-137, ILWL-203 and ILWL-428) tolerant breeding lines (PDL-1 and PSL-9) than in sensitive cultigens (L-4076 and L-4147). Therefore, above wild accessions and tolerant breeding lines appear to be appropriate candidates in terms of regulation of Na^+^ and Cl^-^ to the aerial parts in order to avoid the deleterious effects of salt on plant metabolism as observed in other species [61, 62].

The threshold level of salt stress in hydroponic, condition was saturated and the salt regulation mechanism broken down leading to high rates of transport of Na^+^ and Cl^-^ ions into the shoot. This capacity was saturated at a lower level of salinity in the salt sensitive cultigens as compared to the tolerant wild accessions and salt tolerant breeding lines. A greater degree of salt tolerance was found to be correlated with efficient system for selective uptake of K^+^ over Na^+^ [63]. The selective uptake of K^+^ in contrast to Na^+^ was considered one of the important physiological mechanisms associated with salt tolerance in many plant species [64]. Potassium deficiency always accompanies with sodium toxicity [65]. In the present study, it was also found that the Na^+^ content increased and the K^+^ content decreased, indicating competitive inhibition between the absorption of Na^+^ and K^+^. In tolerant breeding lines and wild accession less increase in Na^+^ was accompanied by marginal decrease in K^+^ concentration as compared to sensitive cultigens (Fig 4a–4d). This suggests that better regulation of K^+^ concentration, induces proper internal maintenance of Na^+^ and Cl^-^ ions concentration. Thus, tolerant breeding lines and wild accessions are able to maintain relatively high K^+^ levels in shoots, and this may act as the major monovalent cationic osmoticum in the presence of external salt. The study also showed that higher K^+^ retention in roots and shoots at seedling and reproductive stages is highly associated with higher seed yield.

Production of higher ROS is well documented under salt stress [66]. In this study, salt stress induced H_2_O_2_ production and lower level of H_2_O_2_ was found in tolerant breeding lines and wild accessions than in sensitive ones (Fig 5). To scavenge H_2_O_2_, the activity of antioxidative enzymes, increases in response to salt stress [67, 68]. In our study, it was found that SOD, APX, GPX and CAT activities were higher in tolerant breeding lines and wild accessions than the sensitive ones. However, CAT activity did not show much increase as compared to other enzyme activities (Fig 6a–6d). The present study is consistent with previous results obtained from different crops [69]. Accumulation of organic solutes and in particular proline, in response to salinity stress is a vital adaptation of plants for survival [18, 70]. However, its role varies according to the species. The accumulation of proline in tolerant wild accessions and tolerant breeding lines ascended rapidly, when the concentration of the NaCl raised to 120 mM NaCl at seedlings stage (Fig 6e), indicating that the adjustment capacity of the tolerant breeding lines and tolerant wild accessions was more pronounced in response to salt stress.

Salinity stress in increased vascular bundle area in tolerant wild accession (ILWL-137) and breeding line (PDL-1). Larger vascular tissue, particularly under limited water availability, is certainly responsible for better transport of water and nutrients [71]. Two types of vascular bundles i.e. in stellar and four patches in cortical region were noticed in both the breeding line and wild types. Similar findings are reported by previous researchers [72]. There was restricted uptake of Na^+^ and Cl^-^ by the thicker epidermis of root along with 3 layers of sclerenchymatous cortex and 8–9 layers of thin wall parenchyma in tolerant breeding line and tolerant wild accession (6–7 layers) when compared to sensitive cultigen (L-4076). The prominent endodermis and pericycle layer in tolerant provides an edge for restricted entry of ions when compared with sensitive ones. A similar result has been found in finger millet [41]. The stem of tolerant breeding line and wild accession had thicker epidermis than the sensitive ones which could help in maintaining turgor pressure of the cells. Whereas in sensitive cultigen, under salinity stress higher penetration of ions through thin and injured epidermis of root along with distorted vascular system unable the proper distribution of ions throughout the plant. There was an apparent change in the overall stellar region under salinity stress for both tolerant and sensitive genotypes in root and shoot of sensitive ones. Reduction in overall cross-sectional area of cortical and stellar region was observed in *Lotus tenuis* under salinity stress has been reported earlier [73]. The cortical cell enlargement was also observed in sensitive cultigens under salt stress. Which could be due to osmotic disturbance.

Ion accumulation in plant tissues has been proposed as a simple explanation for the deleterious effect on seed yield under salt stress. Reduced seed yield under salinity might be due to higher translocation of Na^+^ and Cl^-^ towards reproductive organs. In the present study, the greater reduction in seed yield/plant in sensitive cultigens was associated with higher reduction of pods/plant than those in tolerant breeding lines (Fig 7). The accumulation of toxic ions (Na^+^ or Cl^-^) in the cytoplasm could inhibit metabolic, enzymes. Dua observed higher Na^+^ concentration in roots than shoots in sensitive genotypes, but similar amount in tolerant genotypes [74]. In this study, similar response of Na^+^ concentration in roots and shoots of tolerant breeding lines and sensitive cultigens was observed (Fig 8). The Na^+^ concentration in shoot was negatively associated with seed yield with the tolerant breeding lines having lower Na^+^ concentrations and higher yields than the sensitive cultigens (Table 3). The higher accumulation of Na^+^ in sensitive cultigens might have induced more necrosis in older leaflets and thus shortened duration of individual leaflets and affected the yield [75].

The ranking of genotypes based on seedling survivability in hydroponics at 120 mM NaCl and seed yield in the field and the crucial parameters of Na^+^ and Cl^-^ were significantly correlated. This consistency of ranking between hydroponics and field confirm the reliability of the overall results and suggests that hydroponic screening may be reliable for identifying salt tolerant genotypes. It has been noted that correlation coefficient among all the parameters including seed yield and seedling growth, biomass, salinity scores, Na^+^, Cl^-^ and K^+^ and seedling survivability were analysed by simple correlation. The seed yield in the field was highly correlated with seedling survivability under hydroponics (Table 3). Thus, it is recommended that seedling survivability should be used for screening of large number of genotypes for salinity stress tolerance. Previous findings provide evidence for use of seedling survival as a selection criterion for salinity in cowpea [53]. The wild lentil accessions (ILWL-9 ILWL-95, ILWL-137, ILWL-203, and ILWL-428) were entrusted with better tolerance and may be used as excellent source for improving the tolerance of the cultivated genotypes either through conventional breeding or genetic engineering.

The assessment of salinity tolerant and sensitive genotypes on the basis of germination, seedling growth and biomass accumulation, salinity scores, seedling survivability, Na^+^, Cl^-^ and K^+^ at seedling stage under hydroponics and Na^+^, Cl^-^, K^+^ concentrations, pods/plant and seed yield parameters at maturity under saline field, showed consistency in the salinity tolerance in selected genotypes until maturity. This phenomenon has been observed in number of crops [76]. Similar strategy has been followed in faba bean for the selection of genotypes for salinity tolerance under both hydroponic and field conditions [77]. The finding of one of the study is in contrast to our result, where the tolerant and sensitive genotypes selected at the seedling stage in hydroponics did not change their status during the ontogeny of the whole plant under saline filed conditions [26]. This suggests that the screening of germplasm at seedling stage would provide a suitable clue for initial selection of a large number of germplasm or breeding populations for salinity tolerance.

Genotyping are most useful to accelerate pace of breeding for improving salinity tolerance. Therefore, advancement in molecular component of salinity tolerance in lentil is an utmost requirement. Molecular characterization of genotypes before initiating crossing programme, helps to maximize genetic variation present in breeding population and minimizes the effort for the screening. Through molecular assortment 162 genotypes based on SSR markers have been grouped into 3 major clusters, which were further subdivided into sub-clusters. The genotypes used in this study were characterized at 30 SSR loci. Undoubtedly, these loci could not include all salt tolerant major and minor genes. Evidently, some of the tolerant genotypes were mixed with sensitive ones in the clusters. Similar results were obtained in rice genotypes for salt tolerance by Zeng *et al* [78]. DMRT was compared for all the parameters, cluster 2a was followed by 3b and 2b group of cultigens could be used to select the tolerant genotypes. Similarly, cluster 1b was followed by1a and 3c and could be considered for selecting sensitive genotypes. Further, the tolerant and sensitive genotypes from these groups could be chosen based on the values of salinity score, seedling survivability and other parameters, such as reduction in root and shoot length and their fresh and dry weight under salinity stress. Based on these criteria, PDL-1 and PSL-9 (tolerant breeding lines) and L-4076 and L-4147 (sensitive cultigens) could be chosen as contrasting parents for future breeding programmes for improving of salinity tolerance. On the basis of population structure, also these genotypes are part of different population groups originating from ICARDA (PDL-1) and India (L-4076 and L-4147). Selection of genotypes originating from different area of adaptation will be more useful as their variable evolution history and will provide the hybrid vigour. The genotypes clustered in subgroup 2 a are mostly wild types originating from Mediterranean region and belonged to different *Lens* species *viz*. *L*. odemensis, *L*. orientalis, *L*. nigricans, *L*. *lamottei* and *L*. *ervoides*, of which only, the first two species are crossable. One accession of *L*. *orientalis* (ILWL-95) is salt tolerant and can be easily utilized for transferring tolerance gene(s) into the cultigens. The cultigens were grouped into two separate populations groups, some of which were forming admixtures. Similar distinction of genotypes into populations separating wild and cultigens was also reported by Singh *et al* [33].

## Conclusions

Hydroponic was found a potential technique for evaluation of salt tolerance at seedling stage. The phenotyping traits such as restricted of Na^+^ and Cl^-^ movement coupled with thick epidermis and increased vascular bundles; decreased H_2_O_2_ production, increased K^+^ and proline accumulation, antioxidant enzymes activity, seedling growth, biomass and seedling survivability, pods and seed yield, were found to be associated with salinity tolerance. The regulation of Na^+^ and Cl^-^ in roots and shoots was found performing better in the salt tolerant breeding lines and wild accessions, whereas these ionic regulations were poorly controlled in the salt sensitive cultigens. The uptake of physiological K^+^ concentration by the roots and further translocation and distribution within the plant organs, is also better controlled and integrated with growth in the salt tolerant cultivars as compared to salt sensitive cultigens. The agronomical traits like pods/plant and seed yield/plant, can also be used to distinguish salt-tolerant lentil genotypes from salt-sensitive ones during reproductive stage under saline field conditions. The SSR markers used in the present study were not able to categorise the genotypes on the basis of salt tolerance however, the molecular assortment could help in selecting the contrasting lines with diverse genetic base for further mapping and introgression studies for improving salt tolerant in lentil.

## Supporting information

S1 Fig Phenotypic responses of lentil genotypes under 120mM NaCl. (TIF)

S1 Table Sequence of 30 SSR markers used for molecular scanning. (DOCX)

S2 Table The growth parameters under 120mM NaCl. (XLSX)
