# Correction: Discerning morpho-anatomical, physiological and molecular multiformity in cultivated and wild genotypes of lentil with reconciliation to salinity stress

**DOI:** 10.1371/journal.pone.0190462

**Published:** 2017-12-28

**Authors:** Dharmendra Singh, Chandan Kumar Singh, Shanti Kumari, Ram Sewak Singh Tomar, Sourabh Karwa, Rajendra Singh, Raja Bahadur Singh, Susheel Kumar Sarkar, Madan Pal

Fig 7 is incorrect. Data of the tolerant and sensitive genotypes are incorrect in graphs 2014–15 Pod/Plant (% reduction)/Genotypes, 2013–14 Seed Yield/plant (% Reduction)/Genotypes, and 2014–15 Seed Yield/plant (% Reduction)/Genotypes. The graph 2013–14 Pod/Plant (% reduction)/Genotypes is correct. The data given in the text of the article are correct. The authors have provided a corrected version here.

**Fig 7:**
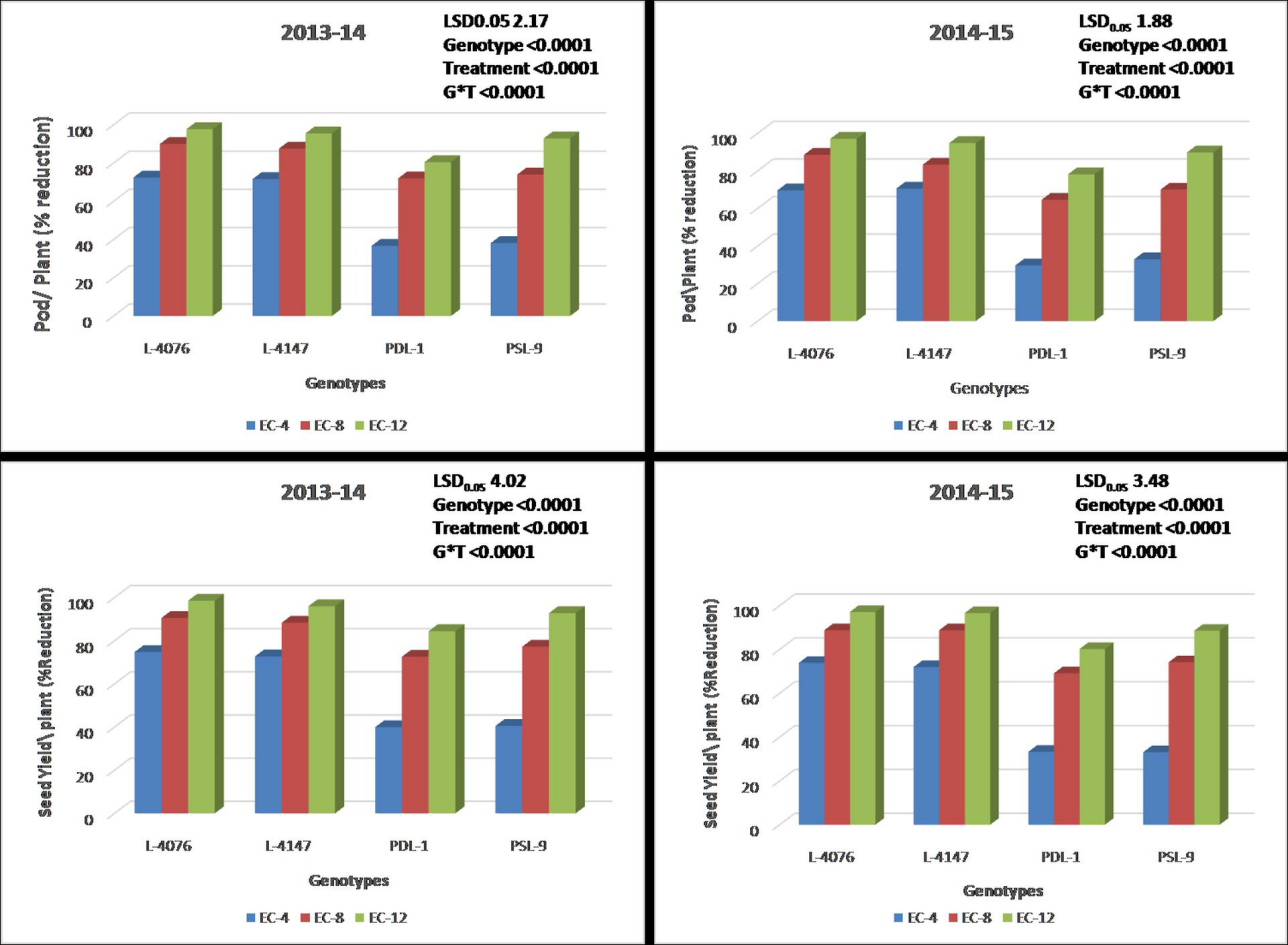
Percent reduction in pods and seed yield per plant in contrasting lentil genotypes grown under three salt concentrations during 2013–14 and 2014–15.
