# Supplementary figures and images for: Discerning morpho-anatomical, physiological and molecular multiformity in cultivated and wild genotypes of lentil with reconciliation to salinity stress
Source: PLoS One. 2017 May 25;12(5):e0177465. doi: 10.1371/journal.pone.0177465 (PMC5444645; doi:10.1371/journal.pone.0177465)

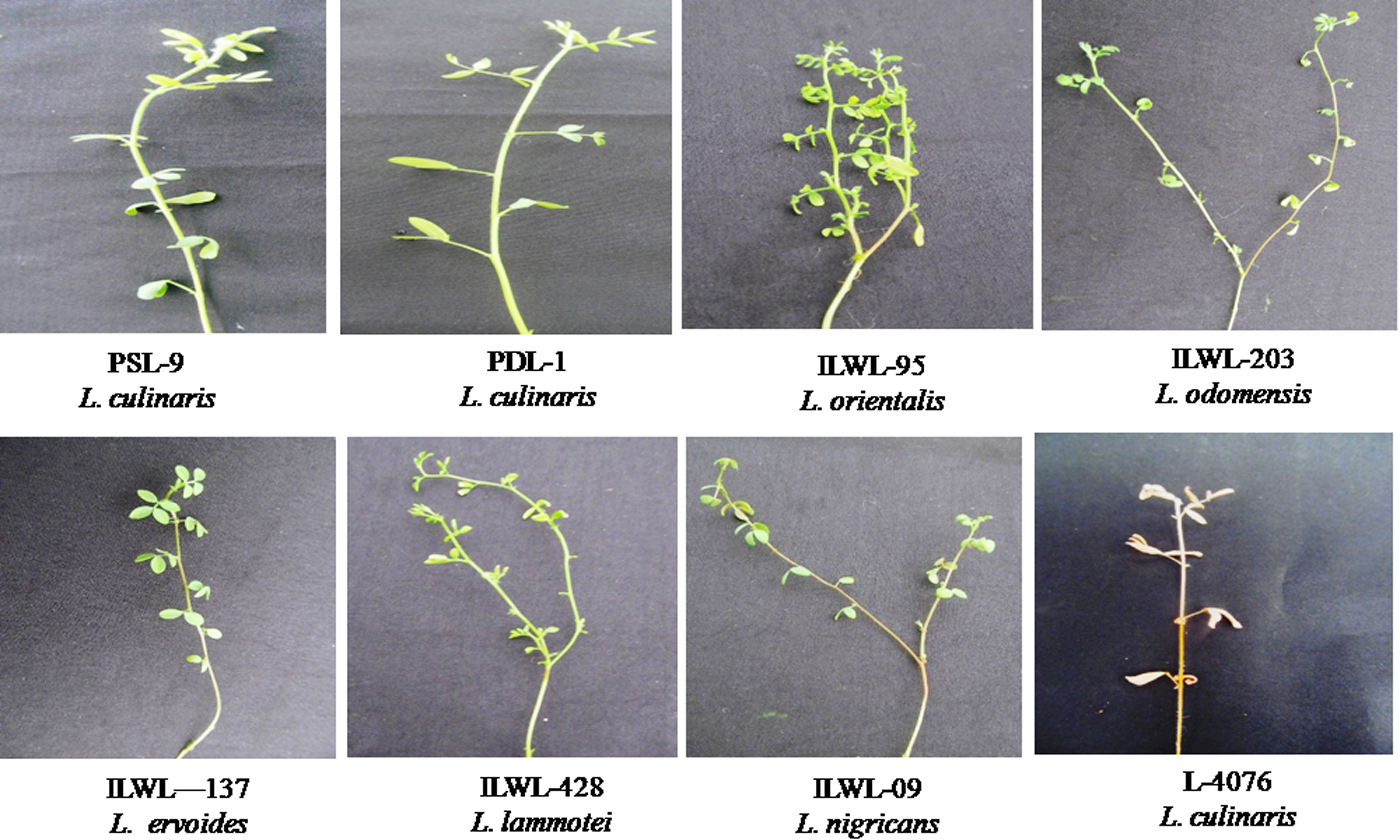

Supplement: S1 Fig — (TIF) [file pone.0177465.s001.tif]
