# Supplementary material for: Discerning morpho-anatomical, physiological and molecular multiformity in cultivated and wild genotypes of lentil with reconciliation to salinity stress
Source: PLoS One. 2017 May 25;12(5):e0177465. doi: 10.1371/journal.pone.0177465 (PMC5444645; doi:10.1371/journal.pone.0177465)
Supplement: S1 Table — (DOCX) [file pone.0177465.s002.docx]

| **S.No.** | **SSR Markers** | **Sequence** |
| --- | --- | --- |
| 1 | LC-01 | FORWARD 5'-GAA ACA ACA CCG AAA TAC AC-3' |
|  |  | REVERSE 5'-CGA AGT CAG ATG AAG TTT G-3' |
| 2 | LC-02 | FORWARD 5'-GAC TCA TAC TTT GTT CTT AGC AG-3' |
|  |  | REVERSE 5'-GAA CGG AGC GGT CAC ATT AG-3' |
| 3 | LC-16 | FORWARD 5'-GAC TCC CAA CTT GTA TG-3' |
|  |  | REVERSE 5'-GTA CAT TGA ACA GCA TCA TC-3' |
| 4 | PBA-LC-117 | FORWARD 5'- GACAACCAAACAGCAACTAAG-3' |
|  |  | REVERSE 5'- TCTGCATCAGACTAGAGCTTC-3' |
| 5 | PBA-LC-118 | FORWARD 5'-GTCTGATGCAGAGAAAACTTC-3' |
|  |  | REVERSE 5'-ACAGTGTTAGTCCCTCCTCTT-3' |
| 6 | PBA-LC-1241 | FORWARD 5'-CTCCATTCTACAACGACTCAT-3' |
|  |  | REVERSE 5'-GTGGGTATCCACCGTAGTTAT-3' |
| 7 | PBA-LC-1247 | FORWARD 5'-GGAAAGAATCGAAATGGAC-3' |
|  |  | REVERSE 5'-GAAGACGGAGCATTGTAGTC-3' |
| 8 | PBA-LC-1308 | FORWARD 5'-TTAGCAAACCCTAATAAGCTG-3' |
|  |  | REVERSE 5'-TACTCCTAGAACATTGGTTGG-3' |
| 9 | PBA-LC-1363 | FORWARD 5'-GGTTAGGTTCCCAAAAGAAG-3' |
|  |  | REVERSE 5'-GGAGTAAGCTCCTCCTCATT-3' |
| 10 | PBA-LC-1401 | FORWARD 5'-AAGCGTAGGTAGACAGAGACA-3' |
|  |  | REVERSE 5'-ACTTTGGTTAGAGCATCAACA-3' |
| 11 | PBA-LC-1698 | FORWARD 5'-GTCCAACAGTTGTAGGAAGTCT-3' |
|  |  | REVERSE 5'-GGAAAGAAGAAGCAGAAGTGT-3' |
| 12 | PBA-LC-216 | FORWARD 5'-AAA TAG AAG TGG AGA GGC AAT-3' |
|  |  | REVERSE 5'-TTC GTT CTT GAG TGA TAT CGT-3' |
| 13 | PBA-LC-221 | FORWARD 5'-AAG CGT CGT AGG TGT AAA AG-3' |
|  |  | REVERSE 5'-GAC TAC TGT GAT GAT GGA TGC-3' |
| 14 | PBA-LC-222 | FORWARD 5'-GAC ACA ACG ACA TCG ATA ATA A-3' |
|  |  | REVERSE 5'-CAT TTA ACA TCT CCG AGT CAC-3' |
| 15 | PBA-LC-368 | FORWARD 5'-ACT ACC AAA GAA GCA GAA AGC-3' |
|  |  | REVERSE 5'-CTG AAT TGC AAA CTT TCT TTG-3' |
| 16 | PBA-LC-376 | FORWARD 5'-TCT CGT TTT CTT CTT CTT CCT-3' |
|  |  | REVERSE 5'-GCG AAG ACG AGT AAG AGT GTA-3' |
| 17 | PBA-LC-377 | FORWARD 5'-TGA ACG AGG TAG AAG AGA CAG-3' |
|  |  | REVERSE 5'-TAG AAA CTC AGA AAC CGA ACA-3' |
| 18 | PBA-LC-379 | FORWARD 5'-CTT TTT CTC TAA CAT CGA AGG-3' |
|  |  | REVERSE 5'-CTT TCA AGC TCG TAA TTG CT-3' |
| 19 | PBA-LC-383 | FORWARD 5'-CAG CAA CAA CTT CCT AAC ACT-3' |
|  |  | REVERSE 5'-GAG TTA GGG TTT GTT TGG TTT-3' |
| 20 | PBA-LC-404 | FORWARD 5'-TCTCTCATCTCTCTCAACCAA-3' |
|  |  | REVERSE 5'-GTTTAGTGACTGGAACCCATT-3' |
| 21 | PBA-LC-652 | FORWARD 5'-GTGGATTAGGGAGTTTGAGTT-3' |
|  |  | REVERSE 5'- CTCCTTCTCAACAGATAACCA-3' |
| 22 | PLC-05 | FORWARD 5'-CAT TGC AGC TTA TTC TCA CAG C-3' |
|  |  | REVERSE 5'-TGA CCC ATC CTC ATC CTT AAA T-3' |
| 23 | PLC-100 | FORWARD 5'-TGC TTT ACT TCC TTC TCT CTT TGC-3' |
|  |  | REVERSE 5'-TAA GCC ATC CAC TTG CAT CC-3' |
| 24 | PLC-104 | FORWARD 5'-AGC TGT TGA TTT TGG CGG-3' |
|  |  | REVERSE 5'-CCG CAG ATC CAG AAA AGA G-3' |
| 25 | PLC-30 | FORWARD 5'-TTG GTC AGG TTC TCA ATC CTC T-3' |
|  |  | REVERSE 5'-ACG GAT GAA CGC TTG TAA AGA A-3' |
| 26 | PLC-35 | FORWARD 5'-TTG CTT CCT CCT CTT CTC ACT C-3' |
|  |  | REVERSE 5'-AGC CTC AGT ACC CTC CTC TTT T-3' |
| 27 | PLC-39 | FORWARD 5'-CAG AGA AAT CCC CTG CTG AG-3' |
|  |  | REVERSE 5'-CAT GAT TCC CAT AGC CTT GC-3' |
| 28 | PLC-51 | FORWARD 5'-CCA TGA TGA GCC TTG AAT GA-3' |
|  |  | REVERSE 5'-TCT TCA ATC TCC AGG AAC ACT TT-3' |
| 29 | PLC-81 | FORWARD 5'-GGG TAG AGT ATT ATT GAA GGT GG-3' |
|  |  | REVERSE 5'-AGA ATC GCT AGT TTA GAG CAA G-3' |
| 30 | PLC-91 | FORWARD 5'-TCA TGG CTT CTA CTT GTA AGG TAA GAC-3' |
|  |  | REVERSE 5'-TCA AAA CCA CAA AAG GAT GGA C-3' |
